# Graphene quantum dot-integrated anodes for lithium-ion batteries: electronic structure, interphase evolution, and multiscale design

**DOI:** 10.1039/d6ra06985a

**Published:** 2026-09-15

**Authors:** Long Di, Qusay Abdulsattar Mohammed, Zeena R. Rhoomi, Man Mohan Shukla, G. Ezhilarasan, Zukhra Yakhshieva, Ibrokhim Sapaev, Vrince Vimal, Gaganjot Kaur, Hoda Kiani

**Affiliations:** a School of Basic Medical Sciences, North Hubei Medical University Wuhan 430000 China; b College of Dentistry, University of Al Maarif Al Anbar 31001 Iraq; c Department of Pharmacy, Ibn Sina University of Medical and Pharmaceutical Sciences Baghdad Iraq; d Department of Computer Science and Engineering, Pranveer Singh Institute of Technology Kanpur UP India; e Department of Electrical and Electronics Engineering, School of Engineering and Technology, JAIN (Deemed to be University) Bangalore Karnataka India; f Chemistry Department, Jizzakh State Pedagogical University Jizzakh City Uzbekistan; g Head of the Department Physics and Chemistry, Tashkent Institute of Irrigation and Agricultural Mechanization Engineers National Research University Tashkent Uzbekistan; h University of Tashkent for Applied Science Uzbekistan; i School of Engineering, Central Asian University Tashkent 111221 Uzbekistan; j Western Caspian University Baku Azerbaijan; k Sharda School of Engineering and Sciences, Sharda University Knowledge Park III Greater Noida India; l Department of Electronics and Communication Engineering, Chandigarh University Mohali Punjab India; m Young Researchers and Elite Club, Tehran Branch, Islamic Azad University Tehran Iran

## Abstract

Graphene quantum dots (GQDs) have emerged as multifunctional nanoscale components for lithium-ion battery anodes, yet their reported benefits are often interpreted without clearly separating intrinsic storage activity from interfacial, conductive, and architectural effects. This review develops a multiscale framework for understanding GQD-integrated anodes through the linked roles of electronic structure, interphase evolution, transport regulation, and electrode organization. It first defines the structural identity of GQDs in terms of confined nanographene domains, edge topology, defect states, dimensionality, and surface chemistry. These features are then translated into functional descriptors governing local reactivity, charge redistribution, interfacial polarization, and coupled ion–electron transport. Experimental evidence across carbon, lithium titanate, metal-oxide, silicon, and silicon-oxide anodes is critically compared to identify recurring structure–property–performance relationships and to distinguish host-dependent GQD functions, including storage-site provision, conductive bridging, interphase regulation, surface protection, and mechanical-contact stabilization. The analysis shows that GQD effectiveness depends less on maximizing defect or dopant content than on matching a controlled GQD configuration to a specific electrode bottleneck. Remaining barriers include uncertain capacity attribution, incomplete interphase characterization, inconsistent material descriptors, limited electrode-level comparability, and manufacturing constraints. A descriptor-guided and failure-aware roadmap is proposed for translating GQD functionality from atomic design to practical anode architectures.

## Abstract 2

This review links GQD design to anode performance, emphasizing host-specific optimization.

## Graphical abstract

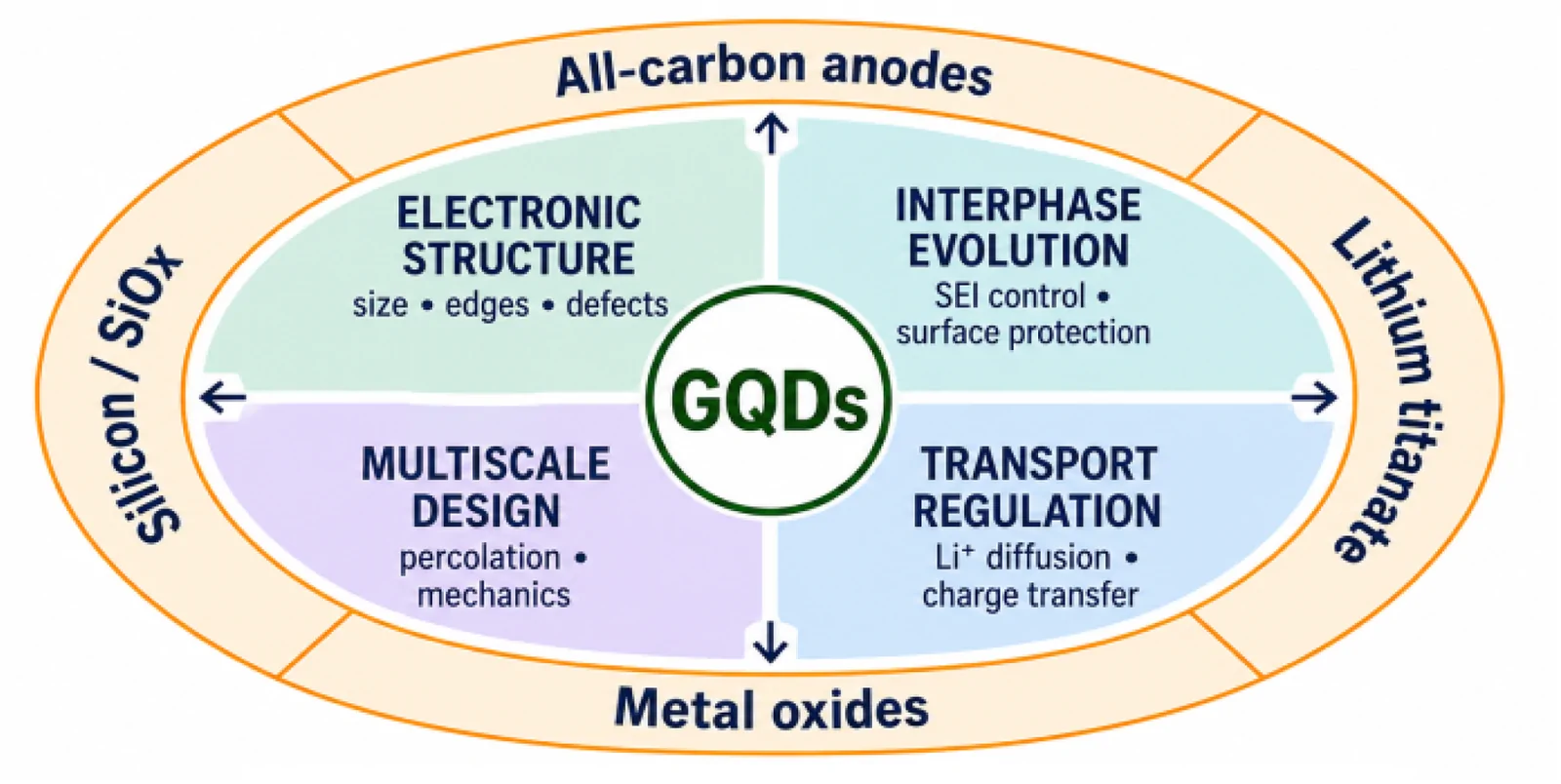

## 1. Introduction

Lithium-ion batteries remain central to portable electronics, electric mobility, and stationary energy storage, but further improvement increasingly depends on anode architectures that can combine high charge-storage capability with fast transport, interfacial stability, and mechanical durability.^1–3^ Conventional graphite provides structural reliability and favourable reversibility, yet its limited storage capacity and rate response restrict its suitability for applications requiring higher energy and power densities.^4,5^ Alternative anode materials, including silicon, transition-metal oxides, and lithium titanate, offer different combinations of capacity, operating potential, and safety, but frequently introduce new limitations such as low electronic conductivity, large dimensional variation, unstable electrode–electrolyte interfaces, sluggish reaction kinetics, or progressive loss of electrical contact.^6–9^ These constraints have shifted attention from the search for a single high-capacity phase toward multicomponent electrode designs in which nanoscale carbon materials regulate transport, interfacial chemistry, and structural evolution.

Graphene quantum dots (GQDs) are particularly relevant to this design transition because they combine a confined sp^2^-carbon framework with a high fraction of edge atoms, chemically adjustable surface states, size-dependent electronic structure, and compatibility with diverse host materials. Unlike extended graphene sheets, GQDs can be distributed across curved surfaces, internal pores, particle boundaries, and nanoscale junctions without necessarily forming large diffusion barriers or restacked layers.^10–12^ Their electronic and chemical characteristics can be adjusted through edge termination, defect regulation, heteroatom incorporation, and surface functionalization. These attributes allow GQDs to operate as lithium-interaction domains, conductive bridges, interfacial polarization centres, protective coatings, or mechanically adaptive connectors. However, the functional role of GQDs is strongly host-dependent, and the same structural feature may generate different consequences in carbonaceous, insertion-type, conversion-type, or alloying anodes.^13–16^

This diversity has created a major interpretive challenge. Improvements attributed to GQDs are often discussed through broad concepts such as enhanced conductivity, increased active-site density, faster ion transport, or structural stabilization.^17,18^ Such explanations rarely establish which atomic configuration produces the relevant electronic state, how that state changes after contact with the host, or whether the observed performance originates from intrinsic GQD activity, interfacial reconstruction, altered porosity, conductive-network formation, or changes in electrode processing. A higher concentration of defects or heteroatoms, for example, may increase local reactivity but simultaneously interrupt π-electron delocalization, promote irreversible reactions, or generate chemically heterogeneous interphases. Similarly, a conformal GQD coating may improve electrical contact while restricting electrolyte access when its coverage becomes excessive.^19–22^ The critical variable is therefore not the mere presence of GQDs, but the compatibility between their atomic configuration, interfacial function, transport environment, and electrode-scale placement.

Previous reviews have provided valuable overviews of GQD synthesis, physicochemical properties, and applications across batteries, supercapacitors, solar cells, and other energy-storage and conversion technologies.^23,24,26,27^ More focused contributions have also summarized the use of GQDs as electrode materials, conductive components, and auxiliary modifiers in batteries and electrochemical capacitors, frequently supported by comparisons of reported electrochemical performance.^25^ Collectively, these studies establish the broad technological relevance of GQDs and identify recurring advantages related to conductivity, surface area, edge activity, and compositional tunability. However, their broad device scope or application-oriented organization leaves the relationships among GQD structural identity, host-specific interfacial function, and electrode-level behaviour insufficiently integrated. In particular, the distinction between lithium stored directly by GQDs and performance gains arising from conductive-network formation, coating architecture, interphase modification, or host-morphology changes remains inconsistently addressed.^23–27^

Within the framework of this review, structure refers to the intrinsic physical and chemical configuration of the GQD, including lateral size, thickness, edge topology, defect distribution, sp^2^-domain continuity, surface functionalization, and dopant bonding configuration. Property denotes the intermediate electronic, chemical, and transport characteristics generated by that structure, such as density of states, Fermi-level position, work function, local charge distribution, Li-binding strength, surface polarity, charge-transfer susceptibility, and ion/electron accessibility. Performance refers to the experimentally observed electrode-level outcomes, including reversible capacity, rate capability, coulombic efficiency, impedance evolution, cycling stability, and retention of electrical and mechanical integrity. These levels are hierarchically connected as structure → property → performance: atomic and nanoscale structural configurations regulate physically meaningful properties, which in turn determine electrochemical behaviour within a given host architecture and operating condition. Accordingly, the term property–performance relationship refers specifically to the link between these intermediate functional descriptors and measurable battery response, whereas structure–property–performance relationship encompasses the complete causal sequence from GQD configuration to electrode-level outcome.

The present review is therefore positioned not as a broader catalogue of GQD applications, but as a focused mechanistic analysis of GQD-integrated lithium-ion battery anodes. It organizes the literature through a multiscale sequence linking nanographene-domain structure, edge topology, defects, layer organization, surface functionality, and dopant configuration to electronic descriptors, interfacial polarization, dynamic interphase evolution, coupled ion–electron transport, and electrode-scale percolation. Evidence from carbonaceous, lithium titanate, metal-oxide, silicon, and silicon-oxide anodes is compared using host-dependent functional categories, including direct storage participation, conductive bridging, interphase regulation, surface protection, and mechanical-contact stabilization. The review additionally examines whether proposed mechanisms are supported by appropriate controls, whether performance remains meaningful at relevant loading and electrode conditions, and which reporting and manufacturing limitations hinder cross-study comparison. This framework clarifies the conditions under which GQD incorporation can provide a mechanistically supported benefit, without implying that GQDs are universally advantageous across all anode architectures.

## 2. Structural identity and property landscape of GQDs

### 2.1. From extended graphene to confined nanographene domains

GQDs should not be defined merely as graphene sheets reduced below an arbitrary lateral-size threshold. Their structural identity emerges from the simultaneous presence of a laterally confined sp^2^-carbon framework, a finite atomic boundary, discrete or quasi-discrete electronic states, and a surface chemistry that does not completely disrupt the graphene-like conjugated core. This definition distinguishes GQDs from extended graphene, graphene oxide fragments, amorphous carbon nanoparticles, and molecular fluorophores that may exhibit similar dimensions but lack an electronically coherent nanographene domain. Accordingly, the transition from graphene to a GQD is not a simple geometric miniaturization. It is a dimensional crossover in which translational symmetry is lost, the continuous band structure becomes discretized, and the proportion of boundary atoms becomes comparable to that of basal-plane atoms.^28–30^ Quantum confinement is therefore necessary but not sufficient for assigning GQD identity; crystallinity, layer organization, edge structure, and the continuity of the π-electron network must also be considered.

An important distinction must also be made between GQDs and the broader class of carbon quantum dots (CQDs). Although both materials occupy the nanoscale carbon-dot regime and may contain mixed sp^2^/sp^3^ bonding, GQDs are characterized by a more structurally coherent nanographene domain in which interconnected sp^2^-hybridized carbon preserves recognizable graphene-like ordering. CQDs, in contrast, generally encompass more structurally heterogeneous carbon nanoparticles containing variable proportions of sp^2^ and sp^3^ carbon, disordered or amorphous regions, and molecular or surface-derived states. This distinction should not be interpreted as implying that all CQDs are completely amorphous or that all GQDs are defect-free crystalline graphene. Rather, the defining difference lies in the extent and continuity of the graphitic sp^2^ framework and the degree to which crystallographic ordering can be experimentally resolved. Consequently, classification based solely on particle diameter is insufficient, particularly because similarly sized GQDs and CQDs can display substantially different electronic structures depending on the continuity of their conjugated carbon domains.

Raman spectroscopy provides an important, although not independently definitive, probe of this structural distinction. The G band reflects in-plane vibrations associated with sp^2^-bonded graphitic carbon, whereas the D band is activated by symmetry-breaking features such as edges, vacancies, structural disorder, and finite-domain boundaries. The commonly reported intensity ratio, *I*_D_/*I*_G_, therefore provides information on disorder and the characteristic organization of sp^2^ domains, but it should not be interpreted simply as a direct measure of either sp^2^-carbon percentage or crystallinity. This consideration is particularly important for GQDs because their very small lateral dimensions inherently generate a high edge-to-basal-plane ratio; consequently, a comparatively intense D band or elevated *I*_D_/*I*_G_ value may coexist with a well-defined graphene-like crystalline core. Changes in *I*_D_/*I*_G_ must therefore be evaluated together with peak position, width, synthesis history, surface functionalization, and complementary structural evidence rather than being used as an isolated criterion for distinguishing highly ordered and poorly ordered carbon dots.

TEM and particularly HRTEM provide the complementary real-space evidence required for this assignment. GQDs are expected to exhibit discernible lattice fringes or ordered nanographene domains when sufficient crystallographic coherence is retained, whereas more structurally disordered CQDs may display weaker, spatially discontinuous, or absent lattice contrast. However, the observation of local lattice fringes alone is also insufficient because heterogeneous carbon-dot ensembles may contain both ordered and amorphous regions. Reliable differentiation should therefore integrate HRTEM-derived lattice ordering with Raman-derived disorder signatures and, where available, spectroscopic assessment of sp^2^/sp^3^ bonding. Such combined characterization provides a more defensible basis for identifying GQDs as confined nanographene structures rather than assigning their identity from nanoscale dimensions alone.

This distinction is especially important because the term GQD is often applied to structurally heterogeneous products whose graphene-like domains, amorphous carbon fractions, molecular species, and oxygenated regions coexist in unknown proportions. Such ensembles may produce averaged spectroscopic signatures that appear compatible with a nanographene structure while concealing major particle-to-particle differences. A rigorous structural description should therefore avoid treating lateral diameter as the sole defining variable. At minimum, GQDs should be represented through a multidimensional identity comprising lateral dimensions, thickness, sp^2^-domain continuity, crystallographic order, edge-to-basal-plane ratio, and chemical composition. The absence of uniform definitions and size criteria across the broader carbon-dot field has contributed to inconsistent structure–property interpretations and has made apparently similar materials difficult to compare.

Conceptually, a GQD is best regarded as a finite nanographene system rather than a generic carbon nanoparticle. Its intrinsic properties originate from how confinement modifies the graphene lattice without completely eliminating its delocalized electronic character.^31–33^ Establishing this structural identity is essential before individual property trends can be assigned to size or chemistry. Once the confined nanographene domain is defined, the atomic structure of its boundaries becomes the next determinant of its electronic landscape.

**Fig. 1:**
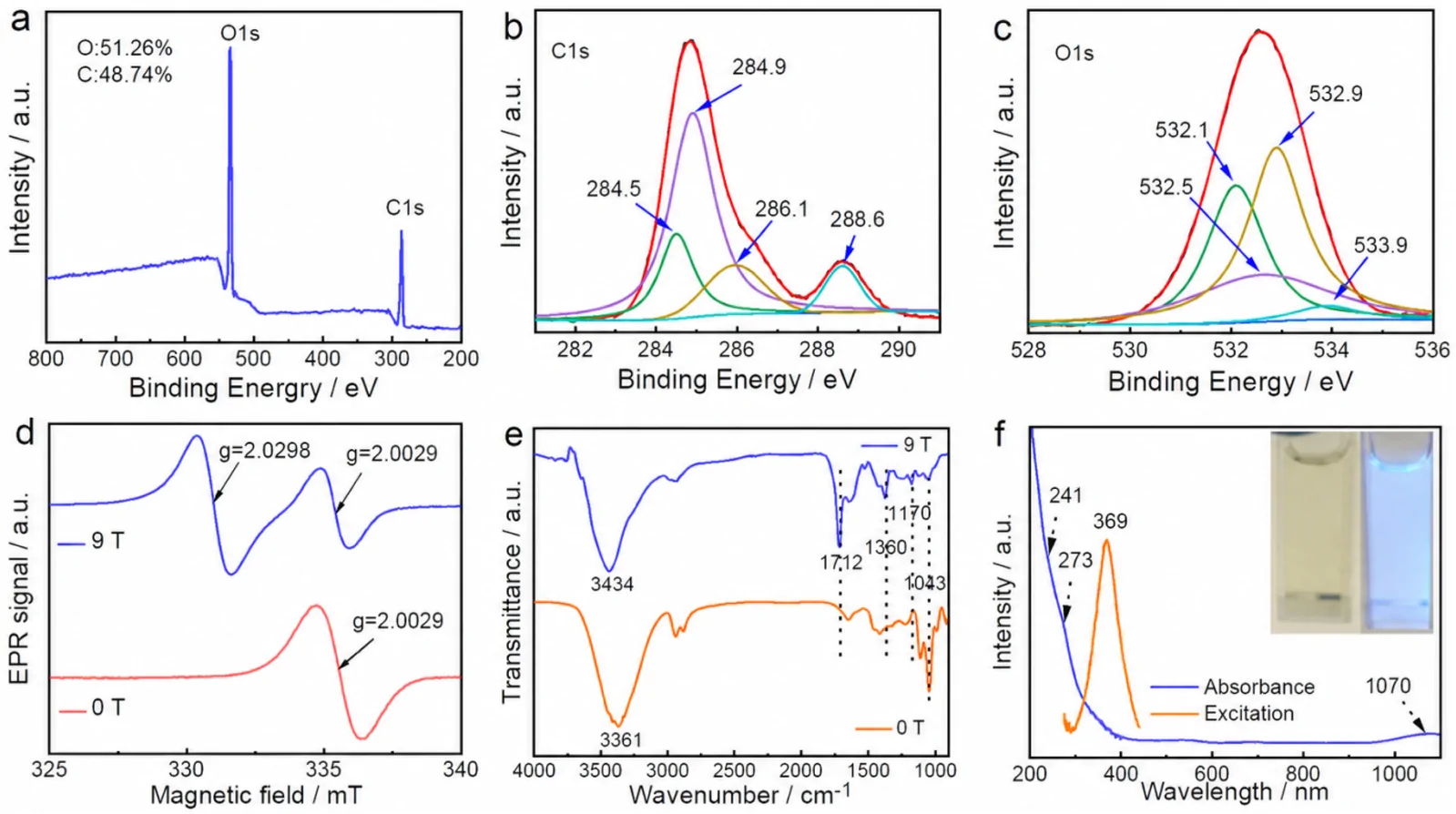
Surface chemical and electronic characteristics of 9T-GQDs. (a) XPS survey spectrum; (b and c) high-resolution C 1s and O 1s spectra; (d) EPR spectra of 9T-GQDs and 0T-GQDs; (e) FT-IR spectra; and (f) UV-vis-NIR absorption and excitation spectra of 9T-GQDs. The combined data show that oxygen-containing functionalities, defect-associated radical states, and extended π-conjugation jointly contribute to the electronic identity of the GQDs. Reproduced with permission from ref. 33. Copyright © 2020 Elsevier B.V.

Fig. 1 provides complementary evidence that the identity of GQDs cannot be reduced to lateral size alone, because their electronic behaviour is strongly influenced by the chemical state of the confined carbon framework. The XPS survey and high-resolution C 1s/O 1s spectra in panels (a–c) reveal a highly oxygenated surface containing C–O, C=O, O–C=O, phenolic, ether, and superoxide-related species, indicating that the nanographene domain is chemically heterogeneous rather than electronically uniform. The EPR response in panel (d) further confirms the presence of carbon-related dangling bonds and defect-associated radical states, while the additional signal observed for 9T-GQDs suggests that oxygen-centred radical species contribute to the electronic-state landscape. FT-IR analysis in panel (e) supports this interpretation by showing stronger carbonyl/carboxyl-related vibrations and altered ether-related bonding compared with 0T-GQDs. Finally, the UV-vis-NIR and excitation spectra in panel (f) demonstrate that these chemical modifications are coupled to the optical response, including pronounced NIR absorption associated with an extended conjugated system and carbonyl-related transitions. Importantly, however, these data primarily establish surface-state and bonding heterogeneity rather than directly proving crystallographic coherence, layer number, or lattice continuity. Therefore, the figure should be interpreted as complementary chemical–electronic evidence for GQD identity, to be considered together with direct structural probes such as HRTEM, AFM, and Raman analysis.

### 2.2. Edge topology, basal-plane defects, and atomic boundary states

The finite boundary of a GQD contributes to its electronic structure rather than serving only as a passive termination of the graphene lattice. In idealized nanographene models, armchair and zigzag edges impose different boundary conditions on the π-electron wavefunction. Armchair segments are generally associated with stronger intervalley mixing and size-dependent energy-level opening, whereas zigzag segments may support electronic states localized near the Fermi level. The resulting electronic structure can therefore depend on the proportion, continuity, orientation, corner geometry, sublattice balance, and chemical termination of these edge motifs. Accordingly, edge topology is more appropriately considered as a spatial arrangement of atomic configurations than as a single armchair-to-zigzag ratio.^34–36^

Real GQDs may deviate substantially from idealized edge structures. Reconstruction, vacancies, dangling bonds, nonhexagonal rings, incomplete passivation, and local strain can introduce additional structural heterogeneity. Basal-plane defects add another level of complexity.^34–36^ Vacancies may interrupt π conjugation and create under-coordinated carbon sites, whereas Stone–Wales transformations alter lattice topology without removing carbon atoms. Depending on their type and location, such defects may influence local charge distribution, structural curvature, and the energetic distribution of electronic states. Their effects should therefore not be assumed to be uniformly beneficial or detrimental, because they depend on defect identity, concentration, position, reconstruction, and interaction with nearby edge structures.

A further interpretive difficulty arises because edge-derived and defect-related electronic states are not always readily distinguishable in ensemble measurements. Observed electronic features may reflect contributions from crystallographic termination, structural disorder, chemical passivation, or combinations of these factors. Moreover, ensemble-averaged characterization may obscure whether defects are uniformly distributed or concentrated within particular particle populations. Reliable structure–property interpretation therefore benefits from descriptors that retain spatial and configurational information, including defect location, edge continuity, sublattice imbalance, and local bonding environment.^37–39^ These considerations also indicate that edge structure and defect configuration should be interpreted together with particle dimensions and layer organization rather than as isolated variables.

Experimental evidence supports the general importance of edge disorder, defect formation, and finite-boundary effects in modifying GQD electronic behaviour; however, several configuration-specific predictions remain insufficiently validated experimentally. These include the quantitative separation of armchair- and zigzag-derived electronic states in chemically heterogeneous GQDs, the direct assignment of individual vacancy or Stone–Wales configurations to specific energy levels, and the extent to which sublattice imbalance or edge continuity independently controls lithium-storage behaviour under operating conditions.

### 2.3. Size–shape–layer coupling in the electronic structure of GQDs

Lateral size is commonly used to describe quantum confinement in GQDs, although a simple inverse relationship between particle dimension and energy gap represents only an approximate trend. In a finite graphene domain, decreasing lateral dimensions can restrict the spatial extent of π electrons and alter the separation between electronic states. However, particles with similar average diameters may still exhibit different electronic structures when their aspect ratios, boundary orientations, symmetries, or sublattice populations differ. Shape should therefore be considered together with size rather than treated only as a secondary morphological descriptor.^40–42^ Different geometries can impose different combinations of confinement length, corner density, edge topology, and sublattice arrangement, making lateral size alone insufficient for describing the electronic structure of a GQD.

Layer number and stacking configuration provide an additional structural dimension. In monolayer GQDs, the electronic states are primarily associated with in-plane π conjugation, boundary conditions, and electron–electron interactions.^40–42^ Additional layers introduce interlayer interactions and dielectric screening, while different stacking arrangements may alter the degree of orbital coupling and electronic symmetry. Consequently, nominal thickness alone does not necessarily capture the structural differences among AB-, ABC-, twisted-, or partially disordered configurations. Interlayer coupling may influence confinement strength, level distribution, charge redistribution, and screening, although the magnitude of these effects depends on the specific structural configuration.^43–45^

Reduced dimensionality can also make distinctions among different electronic energy scales more important. The quasiparticle gap, optical gap, excitonic energy, and HOMO–LUMO separation do not necessarily represent equivalent quantities, particularly in strongly confined structures. This distinction should therefore be considered when comparing theoretical predictions with experimentally inferred electronic or optical gaps.^43–45^

A more defensible structural description consequently treats lateral size, shape, symmetry, layer number, and stacking as coupled descriptors rather than independent variables. Reporting only mean diameter and nominal thickness may overlook structural information that contributes to state localization and energy-level distribution.^43–45^ At the same time, geometry alone does not fully define the electronic properties of realistic GQDs because surface chemistry and heteroatomic incorporation can further modify the confined π-electron network.

Experimental studies broadly support size-dependent confinement and the influence of layer number on GQD electronic properties, but the more detailed predictions concerning shape, symmetry, stacking order, and twist angle remain less systematically validated in battery-relevant GQDs. In particular, experimentally isolating the effects of triangular *versus* hexagonal or irregular geometry, distinguishing AB/ABC/twisted stacking contributions, and quantifying their independent influence on lithium adsorption, charge transfer, and interfacial behaviour remain important unresolved challenges.

**Fig. 2:**
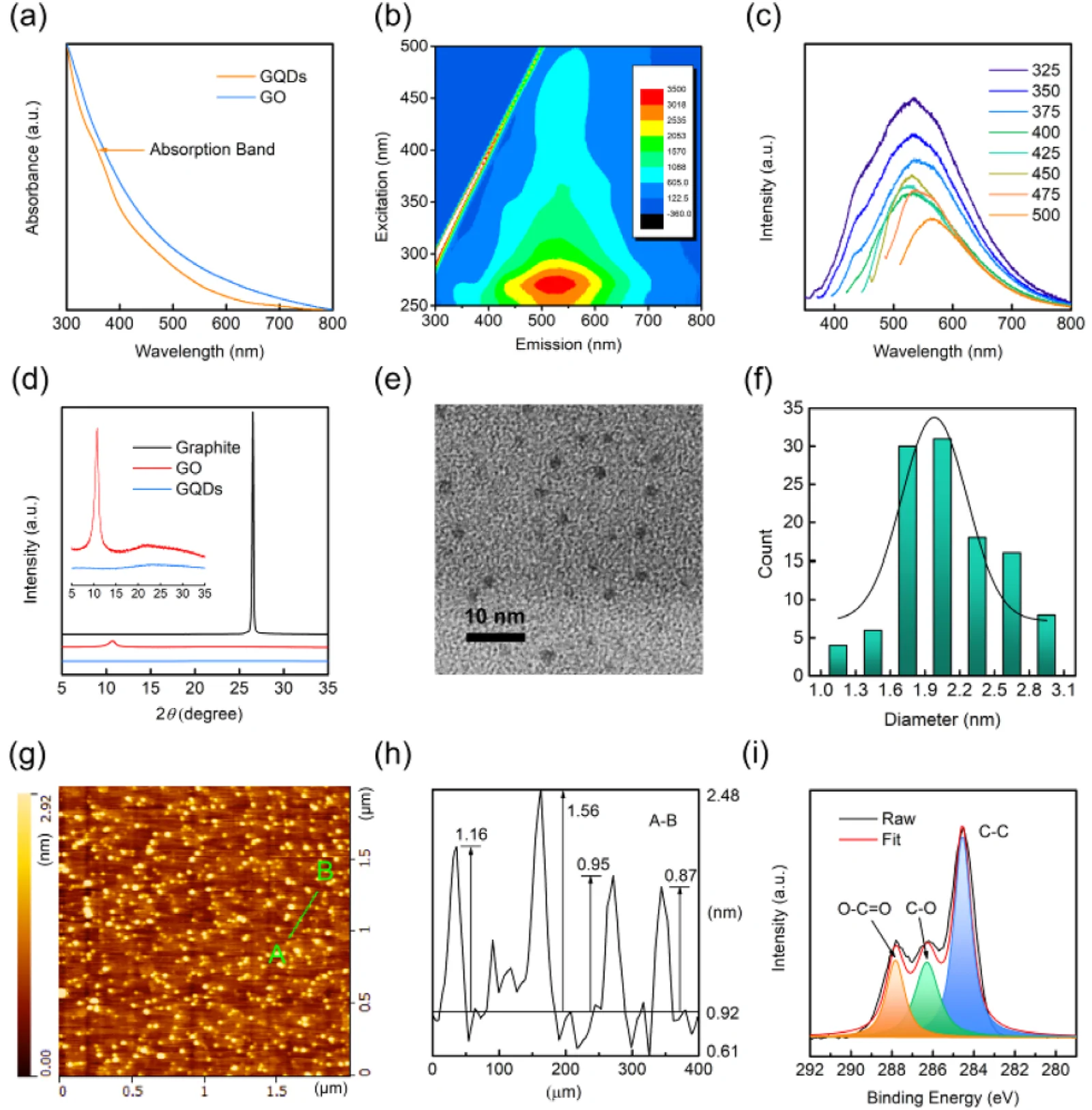
Dimensional, structural, and chemical characterization of GQDs. (a–c) Absorption and excitation-dependent fluorescence; (d) XRD patterns of graphite, graphene oxide, and GQDs; (e and f) TEM image and lateral-size distribution; (g and h) AFM morphology and height profile; and (i) C 1s XPS spectrum. The combined results demonstrate that GQD identity reflects coupled lateral confinement, few-layer thickness, and surface chemistry rather than particle size alone. Reproduced with permission from ref. 44. Copyright © 2020 Elsevier B.V.

Fig. 2 provides complementary experimental evidence for treating GQD size and layer organization as coupled structural descriptors rather than relying on lateral dimensions alone. The absorption and excitation-dependent fluorescence responses in panels (a–c) are consistent with the emergence of confined electronic states after conversion of extended graphene oxide into nanoscale domains, although these optical signatures cannot by themselves distinguish quantum-confinement effects from contributions of surface states. The XRD patterns in panel (d) show loss of the characteristic long-range ordering of graphite and graphene oxide following fragmentation, supporting the transition toward highly confined graphene domains. More direct dimensional information is provided by TEM and statistical analysis in panels (e and f), which reveal narrowly distributed GQDs with an average lateral size of approximately 2.09 nm, while AFM and the corresponding height profile in panels (g and h) indicate thicknesses of approximately 0.8–1.6 nm, corresponding to about 1–3 graphene layers. These observations are particularly relevant because similar lateral dimensions do not necessarily imply equivalent electronic structures when layer number and interlayer coupling differ. Finally, the C 1s XPS spectrum in panel (i) identifies C=C, C–O, and C=O environments, emphasizing an important interpretive limitation: the electronic response cannot be attributed exclusively to size–layer confinement because oxygen-containing surface states coexist with the confined sp^2^ framework.

### 2.4. Surface-state landscapes and heteroatomic chemical diversity

The surface chemistry of a GQD can influence the electronic properties of its confined carbon framework because a substantial fraction of atoms is located at edges or near structural discontinuities. Functional groups such as hydroxyl, carbonyl, carboxyl, epoxide, amine, and thiol groups may alter local hybridization, polarity, charge distribution, and structural planarity. Depending on their bonding configuration and position, these groups can passivate unsaturated sites, introduce localized states, modify local electron density, or partially interrupt π conjugation through sp^2^-to-sp^3^ conversion.^46–48^ Surface functionalization should therefore be considered as one of several variables contributing to GQD electronic structure rather than only as a means of controlling dispersion or solubility.

Heteroatom incorporation further increases structural and electronic heterogeneity. Substitutional, edge-bound, interstitial, and functional-group-associated heteroatoms may produce different effects depending on bonding environment, concentration, and proximity to defects or edges. Nitrogen, for example, can occur in graphitic, pyridinic, pyrrolic, amino, or oxidized configurations, which need not produce identical electronic responses. Similar considerations apply to boron, sulfur, phosphorus, and co-doped systems. Total heteroatom content alone is therefore insufficient for resolving the atomic configurations responsible for a measured response.

The use of a single “surface state” descriptor may also oversimplify the electronic heterogeneity of realistic GQDs. Localized and delocalized states may arise from combinations of edge topology, functional groups, defects, dopants, strain, and solvent-dependent chemical environments.^46–48^ Because ensemble measurements average over these contributions, distinguishing core-derived states from surface- or defect-related states can be difficult. Consequently, apparent continuous tunability in spectroscopic or electronic behavior may partly reflect heterogeneity among different particle populations. Configuration-resolved characterization is therefore preferable when mechanistic interpretation is required.

Among nitrogen configurations, pyridinic, pyrrolic, and graphitic N are expected to contribute through different structure-dependent pathways rather than through a common “N-doping” effect. Pyridinic N is typically associated with edge or vacancy-related environments and can increase local polarity and Li-affinity, thereby favouring adsorption at chemically accessible sites. Pyrrolic N, commonly associated with five-membered rings and defect-rich edge regions, can also enhance local reactivity and lithium binding, but a high fraction of such sites may simultaneously disrupt π conjugation and increase irreversible surface reactions.^47,48^ In contrast, graphitic or quaternary N is incorporated substitutionally within the sp^2^ carbon framework and more strongly preserves electronic delocalization; its contribution is therefore more commonly associated with increased carrier density, improved electronic conductivity, and faster interfacial charge transfer. Amino, oxidized-N, and other edge-bound configurations may further modify surface polarity and electrolyte interactions, but their direct contribution to reversible lithium storage is less consistently resolved.

These configuration-dependent electronic effects can also influence interphase formation. Edge-localized pyridinic and pyrrolic sites may increase the density of initial electrolyte-reaction and SEI-nucleation sites, whereas graphitic N may favour more homogeneous electronic polarization and lower charge-transfer resistance across the GQD–host interface. However, these effects should not be interpreted as a universal ranking of nitrogen species because reported GQD electrodes generally contain mixtures of nitrogen configurations together with simultaneous differences in oxygen content, defect density, particle size, porosity, and host architecture. Accordingly, the present evidence supports a functional distinction rather than an absolute hierarchy: pyridinic and pyrrolic configurations are more closely associated with localized lithium interaction and interfacial reactivity, whereas graphitic N is more strongly linked to electronic transport and charge-transfer regulation. A controlled balance among these configurations is therefore more meaningful than maximizing total nitrogen concentration.

A more rigorous description requires configuration-resolved descriptors, including dopant bonding state, functional-group density and location, sp^2^/sp^3^ ratio, surface charge, dipole heterogeneity, and chemical-state distribution.^49–51^ Together with size, shape, layer organization, edge topology, and defect structure, these variables establish the minimum structural basis needed to interpret GQD behavior. This multidimensional property landscape provides the conceptual foundation for the mechanism-oriented design framework developed in the following section.

The Table 1 establishes GQDs as structurally heterogeneous finite nanographenes whose intrinsic properties emerge from coupled geometric, topological, electronic, and chemical descriptors. It clarifies why isolated metrics such as mean size, dopant percentage, defect density, or oxygen content cannot support reliable mechanistic interpretation. By distinguishing descriptor identity from ensemble-averaged observables, the framework exposes the origins of inconsistent structure–property correlations and defines the minimum information required for configuration-resolved analysis. This multidimensional representation provides a rigorous basis for translating intrinsic GQD characteristics into interfacial, transport, and architecture-level functions.

**Table 1:** Conceptual structure–property map of GQDs: intrinsic descriptors, electronic consequences, and interpretive boundaries

Fundamental dimension	Key structural descriptor	Scientific basis	Intrinsic property consequence	Conceptual limitation	Significance for rational interpretation
Nanographene identity	Continuity of the confined sp^2^-carbon domain	Loss of translational symmetry converts extended graphene bands into discrete or quasi-discrete electronic states	Retention of delocalized π character alongside finite-domain electronic confinement	Small carbon particles may be misclassified as GQDs despite lacking coherent nanographene domains	GQD identity should be established from structural coherence rather than lateral size alone
Lateral confinement	Mean size and size distribution	Reduced lateral dimensions restrict π-electron delocalization and increase electronic-level separation	Size-dependent density of states and confinement-derived energy gaps	Simple inverse size–gap relationships neglect edge chemistry, shape, and structural disorder	Size must be interpreted as one component of a multidimensional descriptor set
Geometric anisotropy	Shape, aspect ratio, symmetry, and corner density	Different geometries impose distinct confinement lengths, sublattice arrangements, and boundary conditions	Redistribution of localized states and modification of level degeneracy	Equal-sized GQDs may possess dissimilar electronic structures because of geometric differences	Shape should be treated as an electronic variable rather than a secondary morphological feature
Layer organization	Layer number, stacking order, and twist angle	Interlayer orbital coupling and dielectric screening modify the electronic states of individual graphene domains	Changes in charge distribution, confinement strength, screening, and state degeneracy	Nominal thickness cannot distinguish AB, ABC, twisted, or disordered stacking configurations	Layer number and stacking geometry should be reported independently
Edge topology	Armchair/zigzag content, continuity, orientation, and corner configuration	Edge motifs impose different boundary conditions on the π-electron wavefunction	Formation of topology-dependent localized or delocalized boundary states	A single armchair-to-zigzag ratio cannot describe edge continuity or spatial arrangement	Configuration-resolved edge descriptors are required for meaningful structure–property analysis
Basal-plane disorder	Vacancy type, Stone–Wales defects, nonhexagonal rings, and reconstruction	Lattice disorder breaks structural or sublattice symmetry and perturbs conjugation	Localized electronic states, charge redistribution, curvature, and possible spin polarization	Defect effects depend on location, reconstruction, concentration, and interaction with edges	Defect density alone is insufficient without information on defect identity and spatial position
Conjugation integrity	sp^2^/sp^3^ ratio and connectivity of aromatic domains	Conversion of sp^2^ carbon to sp^3^ bonding interrupts the extended π network	Trade-off between local chemical reactivity and electronic delocalization	Averaged sp^2^/sp^3^ ratios do not reveal whether disruptions are isolated or network-forming	Interpretation should consider both the amount and spatial continuity of the sp^2^ framework
Surface termination	Functional-group identity, density, location, and protonation state	Surface groups alter hybridization, electronegativity, dipoles, planarity, and local charge density	Passivation, localized-state formation, charge donation or withdrawal, and altered surface polarity	Identical groups can produce different effects at different edge or defect sites	Surface chemistry should be described through site-specific bonding rather than group abundance alone
Heteroatomic diversity	Dopant element, bonding configuration, concentration, and proximity to defects	Substitutional and edge-associated heteroatoms perturb valence-electron distribution and local density of states	Modification of charge density, Fermi-level position, polarity, and electronic asymmetry	Total heteroatom percentage cannot distinguish graphitic, pyridinic, pyrrolic, oxidized, or functional-group-associated states	Dopant configuration is more mechanistically informative than bulk elemental composition
Many-body and screening effects	Dielectric environment, confinement strength, and interlayer screening	Reduced dimensionality weakens electronic screening and amplifies electron–electron and electron–hole interactions	Divergence among quasiparticle gap, optical gap, excitonic energy, and HOMO–LUMO separation	Experimental and theoretical energy gaps may be incorrectly treated as equivalent	The type of measured or calculated energy scale must be explicitly identified
Ensemble heterogeneity	Distribution of sizes, chemical states, defects, and particle subpopulations	Ensemble measurements average signals from structurally non-equivalent GQDs	Apparent continuous tunability may arise from overlapping responses of discrete populations	Mean values can conceal minority populations that dominate electronic or surface behaviour	Statistical distributions and configuration-resolved characterization are essential for reproducible interpretation

## 3. Mechanism-guided multiscale design of GQD-integrated anodes

### 3.1. Descriptor-based translation from electronic structure to local reactivity

The structural variables introduced in the preceding section become relevant to anode design only when they are translated into physically meaningful descriptors. Size, edge topology, defect density, surface termination, and heteroatom configuration do not directly determine electrochemical behaviour; rather, they regulate intermediate properties such as the density of states, Fermi-level position, work function, charge-transfer susceptibility, local electrostatic potential, and binding-energy distribution. A descriptor-based framework is therefore more informative than classifying GQDs solely by synthesis route or elemental composition. It connects atomic structure to reactivity without assuming that a particular modification is universally advantageous.

The central design challenge is that these descriptors are coupled. Increasing defect density may create additional high-affinity sites, but it can also generate deep electronic traps and interrupt the continuity of the π network.^52–54^ Heteroatom incorporation may shift the Fermi level and modify local charge density, yet the direction and magnitude of this shift depend on bonding configuration rather than total dopant concentration. Similarly, oxygen-containing groups can increase local polarity while reducing electronic delocalization through partial conversion of sp^2^ carbon into sp^3^-hybridized domains. Optimizing one descriptor in isolation may therefore deteriorate another.

A useful design principle is to separate descriptors according to their functional roles. Electronic descriptors govern the capacity of a GQD to accept, donate, or redistribute charge. Chemical descriptors determine the strength and reversibility of local interactions. Geometric descriptors control the accessibility and spatial density of reactive regions. These categories should then be evaluated collectively rather than treated as independent optimization targets. The preferred GQD structure is consequently not the one with the highest nominal defect, dopant, or functional-group content, but the one that maintains a controlled distribution of moderately reactive sites within an electronically connected framework.^55–57^ This descriptor-based perspective provides the first bridge from structural identity to functional design. The next question is how these regulated descriptors are modified when GQDs interact with a neighbouring phase and form a real interface.

The three descriptor classes used in this framework are therefore intended as operational rather than purely conceptual categories (Table 2). Electronic descriptors include measurable or calculable quantities such as density of states, Fermi-level position, work function, local charge distribution, and frontier-state separation. Chemical descriptors describe the energetics and identity of local interactions, including Li-binding energy, functional-group density, dopant bonding configuration, surface polarity, and reaction propensity toward electrolyte species. Geometric descriptors quantify the spatial organization of these electronic and chemical environments through lateral dimensions, thickness, edge topology, defect distribution, accessible surface area, and GQD–host contact geometry. Because several quantities are coupled across categories, the classification is functional rather than mutually exclusive; its purpose is to connect experimentally measurable or computationally calculable parameters to specific lithium-storage, charge-transfer, and interfacial processes.

**Table 2:** Operational mapping of electronic, chemical, and geometric descriptors in GQD-integrated anodes

Descriptor class	Operational descriptor	Experimentally measurable quantity/method	Computationally calculable quantity	Relevance to anode behaviour
Electronic	Density of states	UPS/XPS, scanning tunnelling spectroscopy where applicable	Total and projected DOS	Availability and energetic distribution of electronic states for charge transfer and Li interaction
Electronic	Fermi-level position	UPS, Kelvin probe	Fermi energy	Electron-donating/accepting tendency and interfacial charge redistribution
Electronic	Work function	Kelvin probe, UPS	Vacuum-referenced work function	Direction and magnitude of charge transfer at GQD–host interfaces
Electronic	Local charge distribution	XPS chemical shifts, surface-potential measurements	Bader/Hirshfeld charge, electrostatic potential	Identification of electron-rich/electron-deficient Li-interaction regions
Electronic	Electronic gap/frontier-state separation	Optical spectroscopy, STS where available	HOMO–LUMO gap, band gap	Degree of confinement and electronic accessibility
Chemical	Li-binding strength	Indirectly inferred from electrochemical/spectroscopic measurements	Li adsorption/binding energy	Strength and reversibility of Li interaction
Chemical	Surface functional-group density	XPS, FTIR, titration where applicable	Site-specific adsorption/reaction energies	Electrolyte affinity, SEI nucleation and localized Li storage
Chemical	Dopant bonding configuration	High-resolution XPS, XAS	Formation energy and configuration-dependent charge redistribution	Distinguishes graphitic, pyridinic, pyrrolic and other dopant functions
Chemical	Surface polarity/charge	Zeta potential, Kelvin probe	Electrostatic potential, dipole moment	Wetting, ion distribution and interfacial reaction propensity
Geometric	Lateral size and distribution	TEM	Explicit structural model dimensions	Confinement length and edge-to-basal-plane ratio
Geometric	Thickness/layer number	AFM, HRTEM	Monolayer/few-layer structural models	Interlayer coupling and electronic screening
Geometric	Edge topology	HRTEM where resolvable, Raman-supported analysis	Armchair/zigzag fraction and edge configuration	Localization and accessibility of edge-derived reactive states
Geometric	Defect density/distribution	Raman, HRTEM, EPR where applicable	Vacancy density, defect formation energy	Number and spatial distribution of high-reactivity sites
Geometric	Accessible surface/pore structure	BET, porosimetry	Accessible surface and pore descriptors	Ion accessibility and density of electrochemically reachable sites
Geometric	GQD–host contact geometry	Electron microscopy, tomography	Interface area and atomic contact configuration	Electronic connectivity and interfacial reaction-zone density

**Fig. 3:**
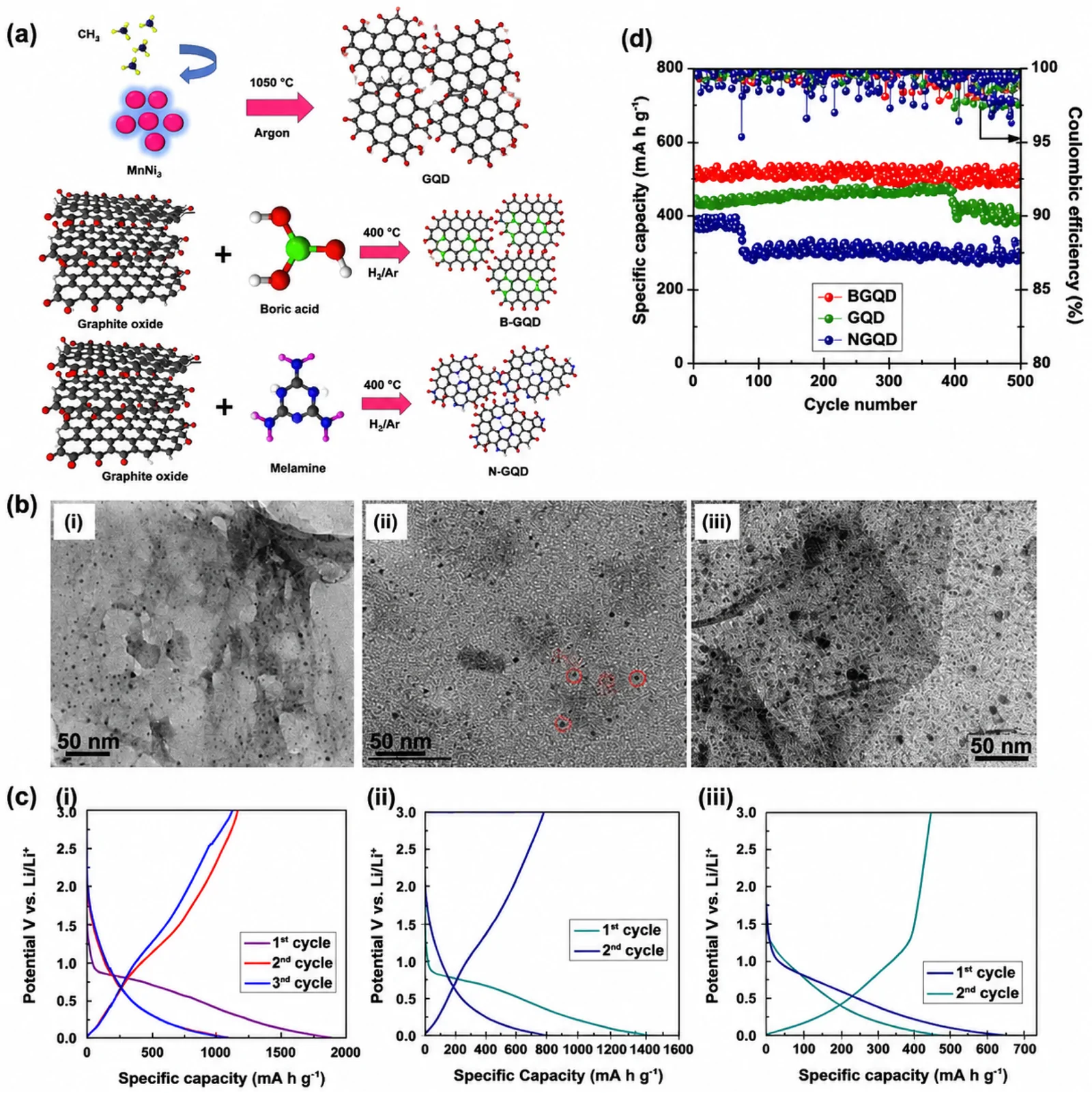
Descriptor-driven structural engineering of GQDs for lithium storage. (a) Schematic preparation of pristine, B-doped, and N-doped GQDs. (b) TEM images of the corresponding GQD structures. (c) Galvanostatic charge–discharge profiles and (d) cycling performance of GQD-based lithium-ion battery anodes. Reproduced with permission from ref. 56. © 2020 Elsevier B.V.

Fig. 3 provides experimental evidence for the descriptor-guided regulation of GQD structures and their influence on lithium-storage behaviour. As shown in Fig. 3(a), different GQD configurations, including pristine GQDs, B-doped GQDs, and N-doped GQDs, can be obtained through controlled structural modification, demonstrating the possibility of tuning the electronic and chemical descriptors of carbon quantum domains. The TEM images in Fig. 3(b) reveal the nanoscale morphology and structural characteristics of the prepared GQDs, confirming the preservation of nanosized graphitic frameworks after heteroatom incorporation. The galvanostatic charge–discharge profiles presented in Fig. 3(c) demonstrate that atomic-level modulation significantly affects lithium-storage capability, with heteroatom-doped GQDs exhibiting enhanced reversible capacity compared with undoped GQDs. This improvement can be associated with the formation of electron-rich and electron-deficient regions within the carbon lattice, which modify local charge distribution and promote Li-ion adsorption and storage. Furthermore, the cycling performance shown in Fig. 3(d) indicates that appropriate electronic regulation can improve reaction reversibility and maintain structural stability during repeated lithiation/delithiation processes. These results highlight that the optimization of GQDs should not rely on maximizing defect or dopant concentration alone; instead, controlled manipulation of electronic, chemical, and geometric descriptors is required to achieve a balanced distribution of reactive sites within an electronically connected carbon framework.

### 3.2. Interfacial polarization and dynamic interphase evolution

The behaviour of GQDs within a composite anode is governed not only by their isolated properties but also by the electronic and chemical reconstruction that occurs after contact with the surrounding matrix. Differences in work function, electronegativity, surface termination, and local bonding can drive charge redistribution across the interface. This redistribution may generate interfacial dipoles, potential gradients, band bending, or localized polarization fields. Depending on their magnitude and spatial distribution, these effects can facilitate charge transfer by reducing energetic discontinuities or, conversely, create trapping regions when interfacial states become excessively deep or electronically disconnected.

An effective interface should therefore be regarded as an electronically active transition zone rather than a geometrically sharp boundary. Its properties depend on contact area, orbital overlap, bonding configuration, lattice mismatch, local strain, and the spatial distribution of defects. Weak physical interactions may preserve the electronic structure of the individual components but provide mechanically or electrochemically unstable contact.^58–60^ Stronger covalent coupling can improve orbital communication while simultaneously perturbing π conjugation or restricting access to chemically active regions. The preferred interfacial configuration is therefore one that maintains sufficient electronic coupling without sacrificing structural adaptability or electrochemical accessibility.

The interface is also dynamically reconstructed during operation. Surface species, solvent-derived products, local charge accumulation, and repeated structural rearrangement progressively transform the pristine contact into a solid-electrolyte interphase (SEI) with its own composition, thickness, ionic/electronic conductivity, and mechanical response. Consequently, characterization of the pristine electrode alone may provide an incomplete representation of the operative interface. Initial SEI growth can passivate highly reactive sites and limit continued electrolyte decomposition, whereas excessive, compositionally heterogeneous, or spatially nonuniform growth may increase interfacial resistance, obstruct ion transport, and intensify local mechanical stress.

GQDs may influence this process through their surface polarity, local charge distribution, functional-group chemistry, and spatial distribution of nucleation sites. However, higher functional-group density should not automatically be assumed to produce a more stable SEI, because chemically heterogeneous GQD surfaces may instead promote nonuniform electrolyte decomposition and repeated interphase reconstruction. The relevant design objective is therefore not suppression of SEI formation, but development of a thin, chemically compatible, ion-permeable, electronically regulated, and mechanically compliant interphase.^61–63^

Direct verification of these proposed functions requires characterization methods capable of resolving SEI morphology, composition, and chemical evolution with minimal perturbation. Cryogenic transmission electron microscopy (cryo-TEM) can preserve metastable and beam-sensitive interphase structures and thereby provide nanoscale information on SEI thickness, continuity, local heterogeneity, and the spatial relationship between GQD domains and interphase products. *Operando* or near-*operando* X-ray photoelectron spectroscopy (XPS) can complement this structural information by tracking potential-dependent changes in carbon, oxygen, fluorine, phosphorus, lithium, and heteroatom bonding environments, helping identify whether particular GQD surface configurations favour specific electrolyte-decomposition products or SEI chemistries. Time-of-flight secondary-ion mass spectrometry (TOF-SIMS), particularly when combined with depth profiling or isotopic labelling, can provide spatially and depth-resolved chemical maps of organic and inorganic SEI fragments and determine whether GQD-rich regions develop compositionally distinct interphases relative to the surrounding host surface. *In situ* Raman spectroscopy can further monitor changes in GQD disorder, conjugation, and local bonding during lithiation and delithiation while correlating these changes with the onset and evolution of surface reactions.

These techniques are most informative when applied to matched GQD-containing and GQD-free electrodes subjected to identical electrochemical histories. Correlating cryo-TEM-derived morphology, XPS chemical-state evolution, TOF-SIMS depth profiles, and *in situ* Raman signatures with impedance growth, coulombic efficiency, and cycling behaviour would provide a more direct basis for determining whether GQDs regulate SEI nucleation and maturation through surface chemistry, electronic coupling, altered electrolyte accessibility, or host-morphology stabilization. Such multimodal characterization is therefore essential for converting the concept of GQD-mediated interphase regulation into an experimentally testable structure–interphase–performance relationship.

### 3.3. Coupled ion–electron transport and reaction-zone accessibility

Transport in GQD-integrated anodes cannot be reduced to either electronic conductivity or ionic diffusivity. Electrochemical reactions require ions and electrons to reach the same spatial region within comparable time scales. An electronically conductive domain that is inaccessible to ions remains underutilized, while an ion-accessible surface connected to a discontinuous electronic network becomes kinetically isolated. The appropriate design target is therefore the density and continuity of coupled reaction zones rather than the independent maximization of conductivity, porosity, or surface area.

GQDs can shorten local electronic pathways and bridge poorly connected domains, but their effectiveness depends on whether these connections form a percolating network. Random dispersion may produce isolated conductive islands, whereas excessive accumulation can reduce open volume and obstruct ionic access. The same trade-off applies to pore development. Small pores increase interfacial area but may restrict solvent-assisted transport or impose high desolvation barriers.^64–66^ Larger channels reduce transport resistance but can lower volumetric contact and weaken structural cohesion. Hierarchical porosity becomes meaningful only when each pore scale performs a distinct transport function and the individual levels remain interconnected.

Reaction-zone accessibility is additionally controlled by tortuosity, pore-neck dimensions, surface charge, wetting behaviour, and local concentration gradients. Total surface area is therefore an incomplete descriptor because it does not distinguish between accessible, partially accessible, and effectively inactive regions. Similarly, bulk conductivity does not reveal whether electronic pathways reach the internal surfaces where reactions occur. Transport analysis should instead focus on effective path length, spatial connectivity, and the overlap between ion-accessible and electron-accessible volumes.

A general design principle is to construct asymmetric but coordinated transport networks. Electrons require continuous, low-resistance pathways through the solid phase, whereas ions require open and wetted pathways through the pore phase. These networks need not share identical geometries, but they must intersect at a high density of stable reaction zones. Mechanical deformation and interphase growth must also be considered because both can progressively alter pore dimensions and pathway continuity.^67–69^ The resulting transport framework shifts attention from static material properties to evolving network accessibility. Translating these nanoscale advantages into a thick and practically relevant electrode, however, requires control over percolation, loading, compaction, and mechanical integrity.

**Fig. 4:**
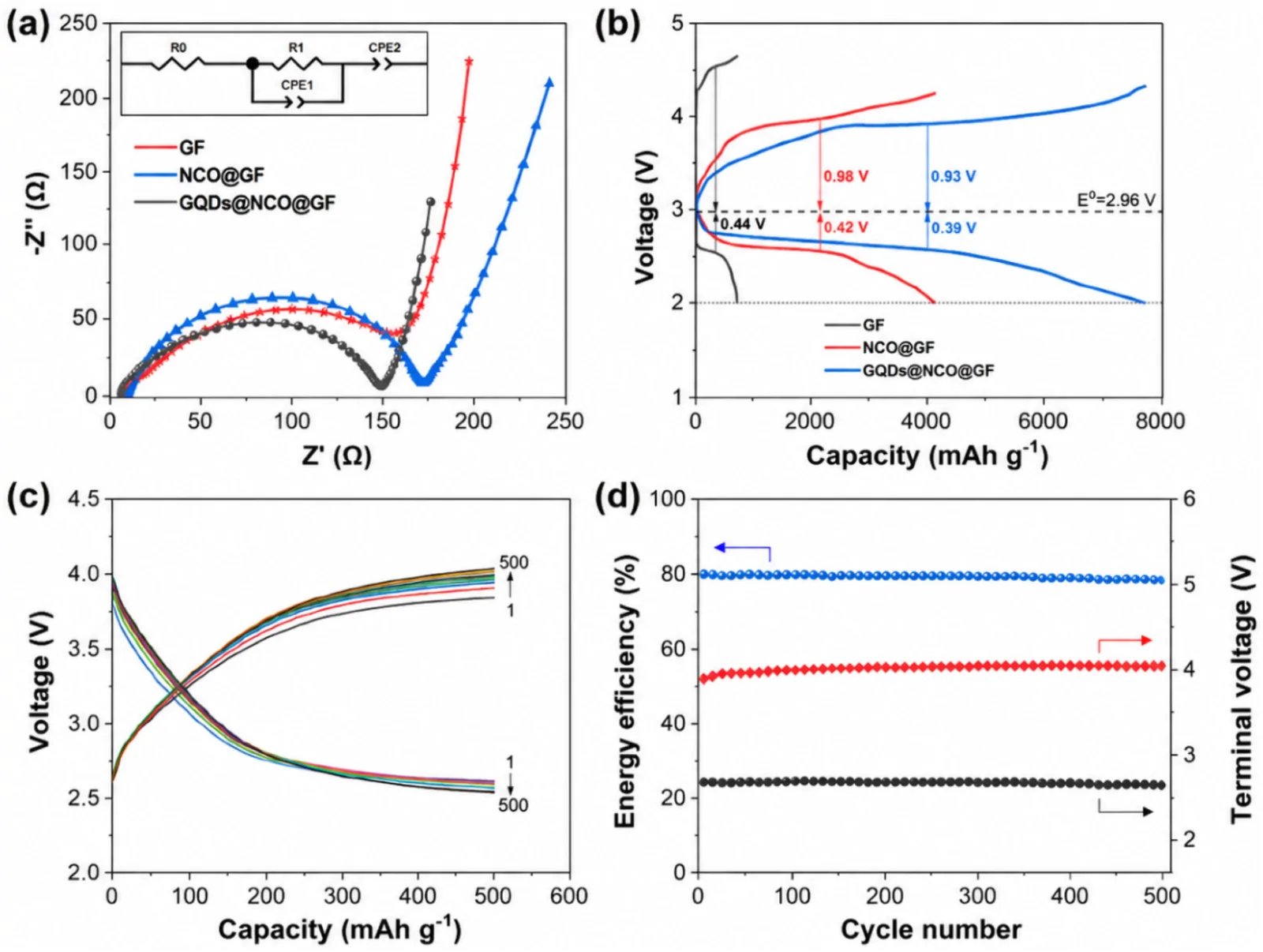
Electrochemical evaluation of GQD-mediated charge transport and reaction-zone accessibility. (a) EIS spectra and equivalent circuit fitting of GF, NCO@GF, and GQDs@NCO@GF electrodes. (b) Discharge–charge profiles at 0.1 mA cm^−2^. (c and d) Cycling performance and corresponding terminal voltage/energy efficiency of the GQDs@NCO@GF electrode. Reproduced with permission from ref. 68. © 2023 Elsevier B.V.

Fig. 4 summarizes the electrochemical evidence demonstrating the role of GQDs in improving coupled ion–electron transport and enhancing the accessibility of active reaction regions. As shown in Fig. 4(a), electrochemical impedance spectroscopy (EIS) measurements reveal a reduced interfacial resistance after GQD incorporation, indicating improved charge-transfer kinetics and more efficient electronic communication between the catalytic phase and electrolyte-accessible regions. The equivalent circuit analysis further suggests that GQD modification decreases the kinetic limitations associated with interfacial charge exchange. The discharge–charge profiles in Fig. 4(b) demonstrate that the GQD-integrated electrode exhibits increased capacity and reduced voltage polarization compared with the pristine and NCO-modified counterparts, reflecting enhanced utilization of electrochemically active domains. This improvement can be attributed to the expansion of effective triple-phase reaction zones, where electronically conductive pathways provided by GQDs are coupled with ion-accessible catalytic interfaces. The repeated discharge–charge curves shown in Fig. 4(c) indicate the preservation of stable reaction behaviour during prolonged operation, suggesting that the regulated transport network can accommodate continuous electrochemical conversion without significant degradation. Furthermore, Fig. 4(d) presents the evolution of terminal voltage and energy efficiency over extended cycling, confirming the capability of the GQD-containing architecture to maintain balanced charge-transfer processes and stable reaction accessibility. Collectively, these results highlight that GQDs function not simply as conductive additives but as nanoscale connectivity regulators that promote the spatial overlap between ion transport pathways and electron conduction networks, which is essential for maximizing the utilization of active regions in composite electrodes.

### 3.4. Electrode-scale translation: percolation, mass loading, and mechanical integrity

The functional value of GQDs is ultimately determined by whether their nanoscale contributions survive electrode fabrication and operation. Atomic regulation, favourable interfacial polarization, and efficient local transport may produce limited benefit when GQDs are unevenly distributed, electronically isolated, covered by inactive components, or concentrated near the electrode surface. Electrode-scale design must therefore translate local functionality into a continuous network extending through the complete thickness of the active layer.

Percolation provides the first constraint. Below a critical connectivity level, GQDs behave as isolated modifiers and cannot support long-range electronic transport. Above an excessive loading threshold, aggregation, pore blockage, increased inactive interfacial volume, and reduced packing efficiency may offset the improvement in connectivity. The design target is a minimally sufficient network that bridges transport discontinuities without becoming a separate densely packed phase. This optimum depends on particle arrangement, aspect ratio, contact resistance, host morphology, and the spatial distribution of conductive additives.^70,71^

Mass loading and electrode thickness introduce further trade-offs. Increasing the active-material quantity improves areal utilization but lengthens ion-transport paths, increases internal resistance, and magnifies reaction heterogeneity across the electrode depth. GQD placement should therefore be spatially programmed rather than assumed to require uniform concentration. Regions near the current collector may benefit primarily from enhanced electronic coupling, whereas outer and internal pore regions may require greater control of wetting, interfacial chemistry, or ionic accessibility.

Mechanical integrity is equally important because transport networks evolve under stress. Particle expansion, contraction, rearrangement, and local fracture can break conductive contacts or isolate pore domains. Strong interfacial adhesion may maintain network continuity, but excessive rigidity can concentrate stress and accelerate delamination. A resilient architecture must combine adhesive contact with sufficient compliance to redistribute deformation. Binder chemistry, compaction pressure, pore volume, and current-collector adhesion therefore become integral design variables rather than secondary processing details.^72,73^

**Fig. 5:**
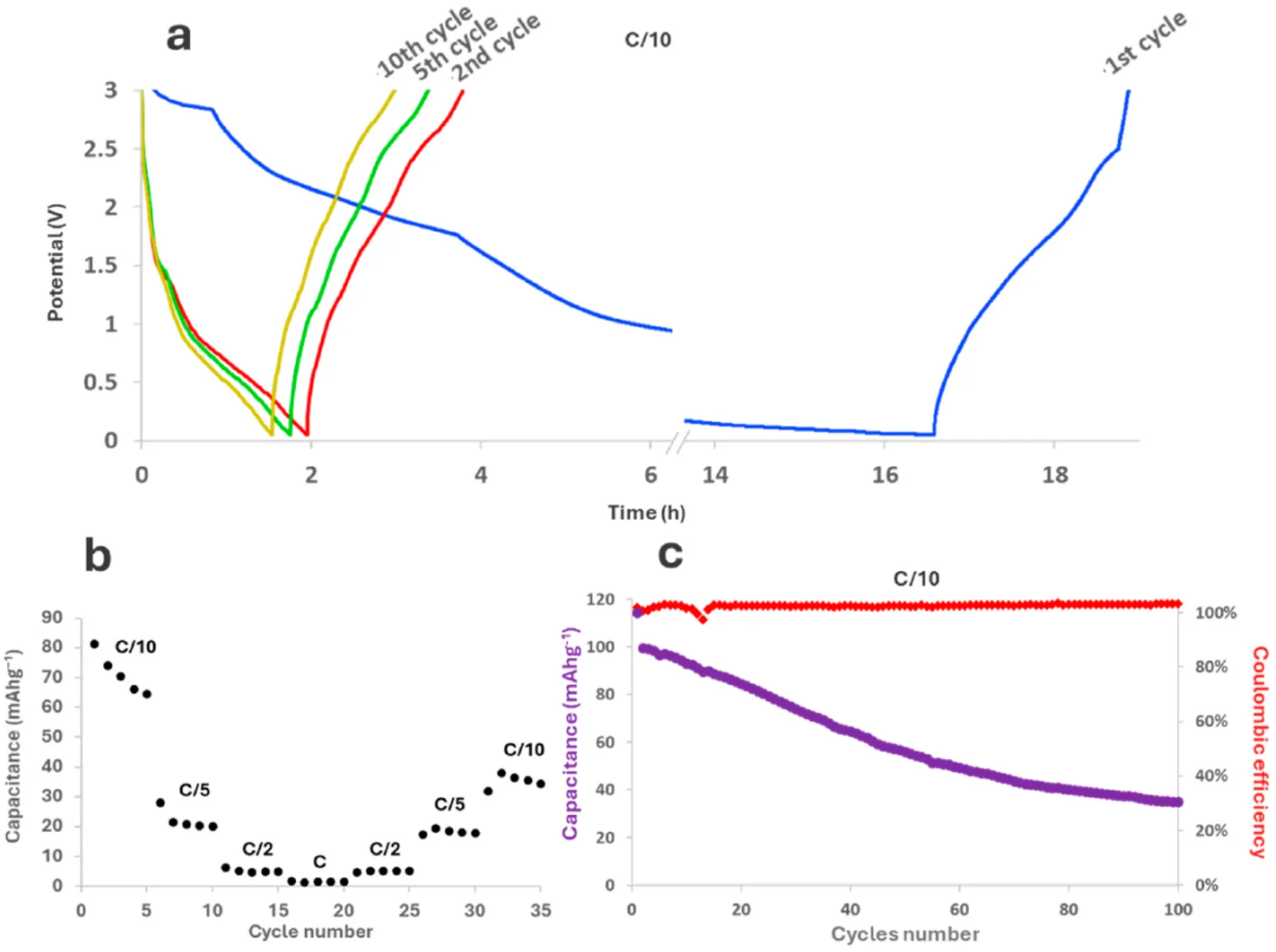
Electrode-scale electrochemical behaviour and degradation mechanisms of GQD-based lithium-ion battery anodes. (a) Galvanostatic charge–discharge profiles at selected cycles. (b) Rate capability under different current densities. (c) Cycling performance and coulombic efficiency. Reproduced from ref. 73 under the Creative Commons Attribution (CC BY) License. © 2024, MDPI.

Fig. 5 provides an electrode-scale perspective on the practical challenges associated with translating the intrinsic electrochemical activity of GQDs into durable anode performance. As shown in Fig. 5(a), the galvanostatic charge–discharge profiles at different cycles reveal distinct electrochemical contributions during the initial operation, where the extended first cycle is associated with irreversible surface reactions and continuous SEI formation. The evolution of the voltage profiles indicates that, although GQDs can participate in reversible lithium storage through their graphitic domains, interfacial instability and incomplete structural cohesion may progressively limit active-material utilization. The rate capability presented in Fig. 5(b) further highlights the dependence of electrode performance on effective transport connectivity, demonstrating that ion accessibility and electronic pathway continuity must be simultaneously maintained to achieve stable operation under different current demands. The cycling behaviour and coulombic efficiency shown in Fig. 5(c) reveal gradual capacity decay despite relatively stable charge efficiency, suggesting that mechanical adhesion, active-material retention, and persistent interfacial reconstruction remain critical limitations at the electrode scale. These observations emphasize that the successful implementation of GQDs requires more than intrinsic lithium-storage capability; it depends on controlling percolation pathways, strengthening electrode-level integration, and preventing progressive loss of electrical contact during repeated cycling. Therefore, this example illustrates the importance of failure-aware design strategies, where GQD distribution, anchoring, and interaction with complementary active components must be optimized to preserve transport networks and structural integrity throughout long-term operation.

The most transferable electrode-level principle is failure-aware integration. GQD concentration, distribution, and anchoring should be selected according to the dominant anticipated failure pathway, whether contact loss, pore collapse, interphase thickening, aggregation, or mechanical separation. This multiscale framework thus links electronic descriptors, interfacial evolution, coupled transport, and electrode integrity without presuming a specific material formulation. The experimental validity of these design relationships can subsequently be assessed through the comparative evidence examined in the following section.

## 4. Cross-platform evidence and performance–mechanism mapping in GQD-integrated lithium-ion battery anodes

### 4.1. Intrinsic storage activity and conductive-network integration in all-carbon anodes

The available evidence indicates that the contribution of GQDs to all-carbon anodes extends beyond increasing the nominal carbon surface area. The computational investigation of lithium-ion and lithium-atom adsorption and their packing on GQDs provides a theoretical basis for treating confined graphene domains as potential storage-active components rather than exclusively as conductive additives. This perspective is experimentally supported by the freestanding GQD-coated carbon-nanotube architecture, in which the GQDs supplied chemically active storage regions while the three-dimensional CNT framework maintained electronic continuity and limited GQD aggregation. The resulting electrode delivered 700 mAh g^−1^ after 100 cycles at 100 mA g^−1^ and retained 483 mAh g^−1^ after 350 cycles at 1000 mA g^−1^.^74^

The structure–property relationship in this system is based on functional separation between the two carbon components. The CNT scaffold primarily provides macroscopic conductivity, mechanical support, and interconnected porosity, whereas the GQD coating contributes short-range electronic states and oxygen-functionalized sites. The reported importance of oxygen-containing groups suggests that the storage response cannot be attributed to quantum confinement alone. Instead, confinement, surface functionality, and nanoscale contact operate together.^27,56^ The coaxial arrangement is particularly relevant because it places the functionalized GQDs directly on a conductive pathway, reducing the likelihood that chemically active domains become electronically isolated.

Nevertheless, the study does not fully separate capacity arising from reversible lithium storage at GQD sites from contributions associated with the CNT framework, interfacial regions, or surface reactions. The freestanding and binder-free configuration also introduces architectural advantages that complicate attribution of the measured performance solely to GQDs. In parallel, the computational study defines the possibility of lithium adsorption and packing but does not, from the supplied information, establish how realistic edge disorder, functional-group heterogeneity, or electrolyte-derived species alter those interactions.^67,75^ Thus, all-carbon evidence supports the active participation of GQDs, but quantitative identification of their intrinsic storage contribution remains unresolved. This attribution problem becomes more explicit when GQDs are used not as the primary storage phase, but as interfacial modifiers of a distinct anode material.

### 4.2. Interfacial charge-transport control in lithium titanate anodes

Lithium titanate systems provide comparatively clear evidence that GQDs can act as interfacial charge-transfer regulators without serving as the dominant active phase. Two distinct modification strategies produced substantial improvements: an N-functionalized GQD interfacial layer and a hybrid containing N,S-co-doped GQDs. In the first configuration, the GQD layer increased the lithium-ion diffusion coefficient by approximately 19%, enabled a capacity of 161 mAh g^−1^ at 50C, and maintained operation for more than 500 cycles.^34,76^ The improvement was associated with simultaneous charge-transfer enhancement, formation of a thin and smooth interphase, suppression of electrolyte-driven surface reactions, and mitigation of gas generation.

The co-doped configuration followed a related but not identical performance pathway. The interconnected porous LTO/N,S-GQD framework delivered 254.2 mAh g^−1^ at 0.1C and 126.5 mAh g^−1^ at 10C, while retaining at least 96.9% of its capacity after 2000 cycles at 2C.^77^ Importantly, the GQD component was reported to improve electron transfer and lithium-storage capacity without substantially obstructing electrolyte transport. This outcome indicates that the nanoscale additive did not merely increase conductivity; it preserved ionic accessibility while modifying the electronic environment of the LTO network.

Taken together, the two studies support a shift from viewing GQDs as passive conductive carbon toward considering them multifunctional interfacial phases. Nitrogen functionalization primarily appears to regulate surface protection, interphase uniformity, and high-rate charge transport, whereas N,S co-doping combines electronic modification with additional storage-related contributions in a porous hybrid structure.^70,77^ However, the evidence does not establish that co-doping is intrinsically superior to single-element functionalization. The two systems differ in synthesis, morphology, GQD incorporation mode, testing rate, and electrode organization, preventing a direct ranking of dopant strategies.

A further limitation is that bulk dopant composition does not reveal which nitrogen- or sulfur-bonding configurations dominate the observed response. The studies demonstrate performance consequences but leave the active atomic motifs insufficiently resolved. Nevertheless, the long-duration retention and high-rate behaviour indicate that interfacial regulation is particularly effective when the host is structurally stable but electronically and kinetically limited.^27,67^ The next group of studies tests whether the same approach remains effective for metal-oxide systems undergoing more extensive structural and interfacial changes.

**Fig. 6:**
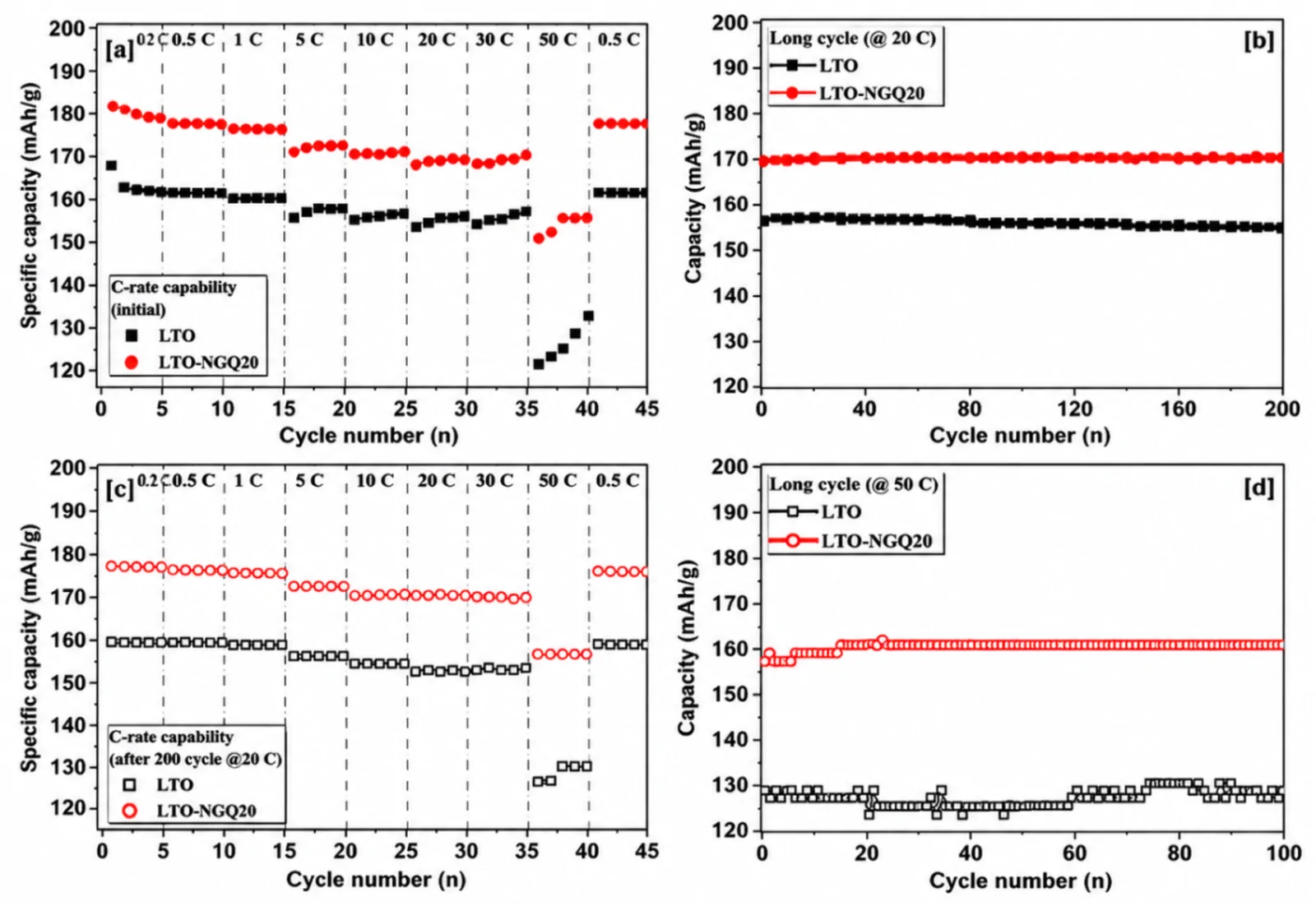
Electrochemical performance of pristine LTO and N-GQD-modified LTO electrodes. (a) Rate capability at different current rates. (b) Long-term cycling performance at 20C. (c) Repeated rate capability. (d) High-rate cycling stability at 50C. Reproduced with permission from ref. 76. © 2019 Elsevier B.V.

Fig. 6 demonstrates the effect of N-GQD incorporation on the high-rate capability and long-term stability of lithium titanate anodes, highlighting the role of GQDs as interfacial charge-transfer regulators. As shown in Fig. 6(a), the LTO-NGQ20 electrode exhibits enhanced rate capability compared with pristine LTO, maintaining higher capacities under increasing current densities up to 50C. This improvement can be attributed to the N-functionalized GQD layer, where electron-rich nitrogen configurations facilitate charge transfer between the LTO active phase and the conductive network, thereby accelerating Li^+^ insertion/extraction kinetics. The long-term cycling behaviours presented in Fig. 6(b) and (d) further reveal that the GQD-modified electrode maintains stable capacity retention under high-rate conditions, indicating improved reaction homogeneity and preservation of conductive pathways during repeated operation. The repeated rate capability evaluation in Fig. 6(c) confirms the reversibility of the enhanced transport network, demonstrating that the N-GQD interfacial layer enables rapid and sustained electrochemical responses without compromising structural stability. Collectively, these results indicate that GQDs function beyond conventional conductive additives by creating an electronically coupled interfacial region that improves charge-transfer kinetics, promotes ionic accessibility, and stabilizes the electrochemical operation of LTO-based anodes.

### 4.3. GQD-mediated stabilization and charge coupling in metal-oxide anodes

Metal-oxide composites provide evidence that GQDs can simultaneously influence conductivity, structural retention, interfacial stability, and utilization of redox-active domains. In the hierarchical CuO system, coating nanoflake-based microspheres with carboxyl-functionalized GQDs reduced charge-transfer resistance, enhanced conductivity, and suppressed dissolution and agglomeration. The composite retained 609 mAh g^−1^ after 200 cycles at 0.2 A g^−1^, whereas the unmodified CuO electrode retained only 61 mAh g^−1^ under the same stated condition. At 2 A g^−1^, the GQD-containing electrode maintained 79.4% of its capacity after 1000 cycles, compared with 0.7% for pristine CuO, while the initial coulombic efficiency increased from 75.2% to 88.2%.^78^

These differences are too large to be explained by conductivity enhancement alone. The GQD coating appears to stabilize the active morphology and reduce formation of an unstable interphase, thereby preserving access to CuO during extended cycling. In contrast, the manganese vanadate/GQD composite was designed around nanoscale coupling between a redox-active oxide and a charge-transporting carbon phase. The resulting anode maintained a discharge capacity of 970 mAh g^−1^ over 100 cycles with coulombic efficiencies close to 100%.^79^ Here, the key structure–performance relationship is the short separation between charge-storage clusters and conductive GQD domains, achieved through a bottom-up fabrication route.

The two studies therefore represent different forms of GQD integration. In the CuO electrode, GQDs behave mainly as a conformal protective and conductive coating around a hierarchical host.^78^ In the manganese vanadate system, GQDs are incorporated as part of a chemically assembled nanoscale composite that links storage and transport components.^79^ Both approaches improve utilization of metal-oxide phases, but the supplied results do not permit separation of the GQD effect from the influence of hierarchical morphology, binder-free construction, current-collector architecture, or precursor-derived nanostructuring.

The comparison also reveals a broader reporting gap. Strong gravimetric capacity and cycling data are available, but the relative mass fraction of GQDs, their direct contribution to stored charge, and the evolution of the oxide–GQD interface are not specified in the supplied summaries. Consequently, GQD-mediated stabilization is well supported at the electrode level, whereas its exact chemical and mechanical origin remains only partially resolved. Silicon-derived systems provide a further test because their performance is dominated by large structural changes and strong dependence on surface chemistry.^67,70^

**Fig. 7:**
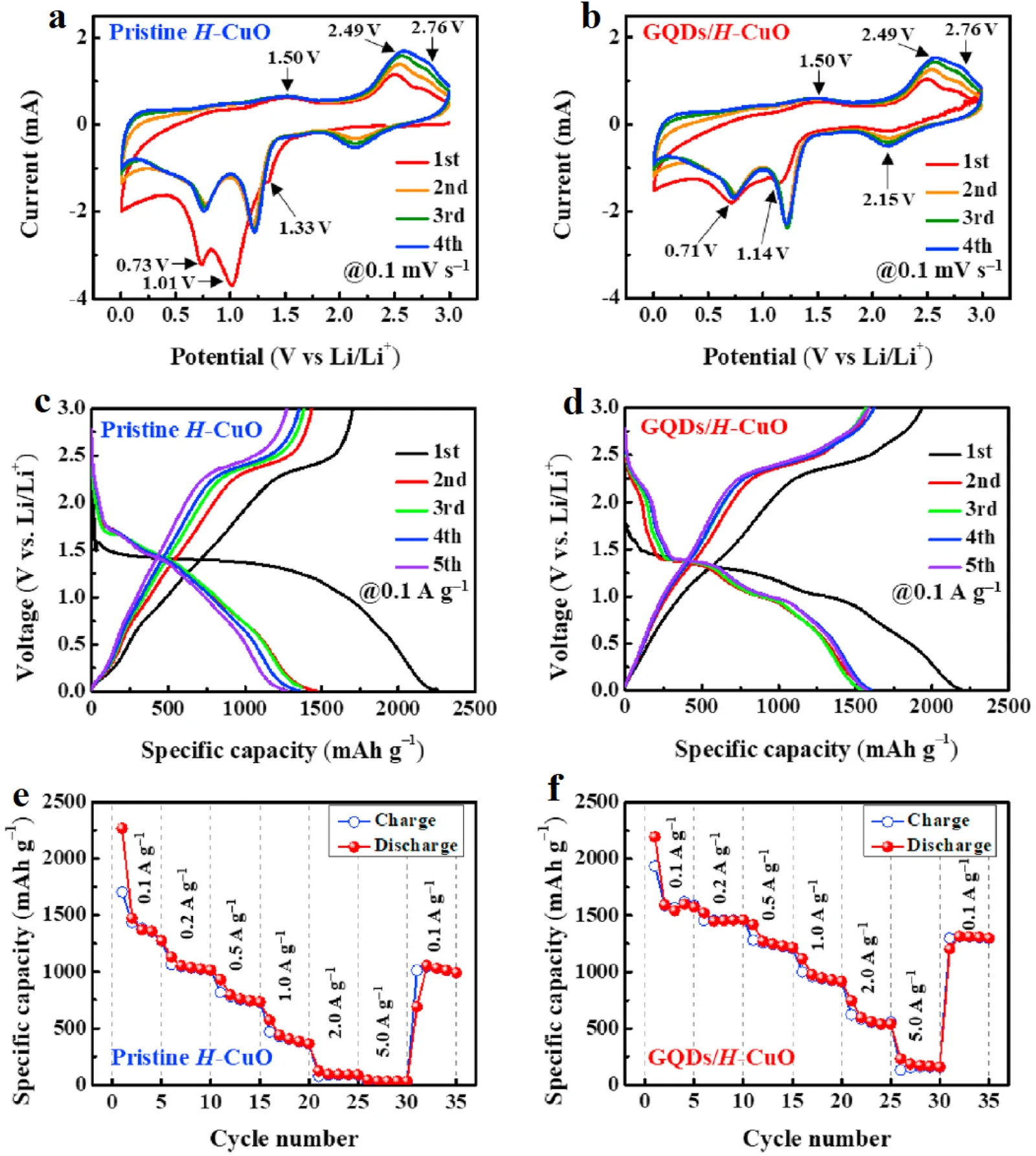
Electrochemical performance of pristine H-CuO and GQD/H-CuO composite anodes. (a and b) Cyclic voltammetry curves. (c and d) Galvanostatic charge–discharge profiles. (e and f) Rate capability at different current densities. Reproduced with permission from ref. 78. © 2021 Elsevier B.V.

Fig. 7 presents the electrochemical response of H-CuO and GQD-modified H-CuO composite anodes, highlighting the role of GQDs in regulating charge-transfer kinetics and stabilizing metal-oxide conversion reactions. As shown in Fig. 7(a and b), the cyclic voltammetry profiles reveal that GQD incorporation improves the reversibility of the multistep CuO conversion reactions, with more stable redox peaks maintained during repeated cycles compared with pristine H-CuO. This behaviour indicates that the GQD layer facilitates more efficient reaction kinetics and preserves electrochemically active interfaces during lithiation/delithiation processes. The galvanostatic charge–discharge profiles in Fig. 7(c and d) further demonstrate reduced polarization and improved voltage-profile stability for the GQD-containing electrode, confirming enhanced reaction reversibility and more effective utilization of CuO active domains. The rate capability results in Fig. 7(e and f) show that the GQD/H-CuO composite maintains higher capacities under increasing current densities, reflecting improved electron transport and electrolyte accessibility provided by the conductive and buffering GQD framework. Collectively, these results demonstrate that GQDs function as multifunctional interfacial regulators in metal-oxide anodes by simultaneously enhancing charge-transfer pathways, limiting excessive interfacial degradation, and maintaining structural integrity during repeated conversion reactions.

### 4.4. Silicon-derived anodes and cross-system design lessons

The silicon-based studies show that GQD effectiveness is sensitive not only to coating coverage but also to the molecular identity of the GQD edge and the composition of the surrounding carbon framework. Phenylalanine-functionalized GQDs coated onto silicon nanoparticles produced capacities of 4066, 3796, and 1820 mAh g^−1^ at 50, 100, and 1000 mA g^−1^, respectively, and retained 3068 mAh g^−1^ after 100 cycles at 100 mA g^−1^.^80^ Comparison with alanine-functionalized GQDs provides one of the more informative controls in the selected literature because it directly links edge chemistry to electrochemical behavior. The phenyl-containing structure was associated with a more favourable energy-level distribution, improved steric organization, and faster electron and electrolyte transport.

The GQD/SiO_*x*_/carbon-nanoparticle system presents a more composition-dependent response. Carbon nanoparticles generated internal cavities intended to accommodate SiO_*x*_ volume variation, while the SiO_*x*_ fraction was varied over a broad range. The composition containing 15 wt% SiO_*x*_ delivered an initial discharge capacity of 595 mAh g^−1^, 92% capacity retention, and a rate-performance ratio of 81% at 2C relative to 0.1C.^81^ However, low initial efficiency remained evident, and SiO_*x*_ contents above 30 wt% resulted in reduced cycling stability. These outcomes show that adding a higher-capacity phase does not produce a monotonic improvement because structural buffering and transport continuity become insufficient beyond an optimum composition.

A direct numerical comparison between the two silicon systems would be misleading. One uses silicon nanoparticles with a chemically tailored GQD coating, whereas the other combines SiO_*x*_, GQDs, and carbon nanoparticles with composition-dependent cavity formation. Their current conventions, active phases, and structural designs differ substantially. Nevertheless, together they reveal two complementary principles: GQD edge chemistry can regulate interfacial transport and surface protection, while electrode composition must preserve sufficient conductive and mechanically accommodating volume.^54,80^

Across all eight references, GQDs perform three recurring roles: they provide additional lithium-interaction sites in all-carbon structures,^75^ regulate interfacial transport and surface reactions in stable host materials,^77^ and preserve conductive contact and morphology in structurally evolving oxides and silicon-derived phases.^79^ The principal unresolved issue is quantitative attribution. The literature rarely distinguishes charge stored directly by GQDs from improvements caused by coatings, porosity, current-collector configuration, or host morphology. Standardized reporting of GQD loading, bonding configuration, initial efficiency, areal capacity, electrode density, and interfacial evolution would therefore be necessary to convert these system-specific improvements into broadly testable performance–mechanism relationships.^81^

**Fig. 8:**
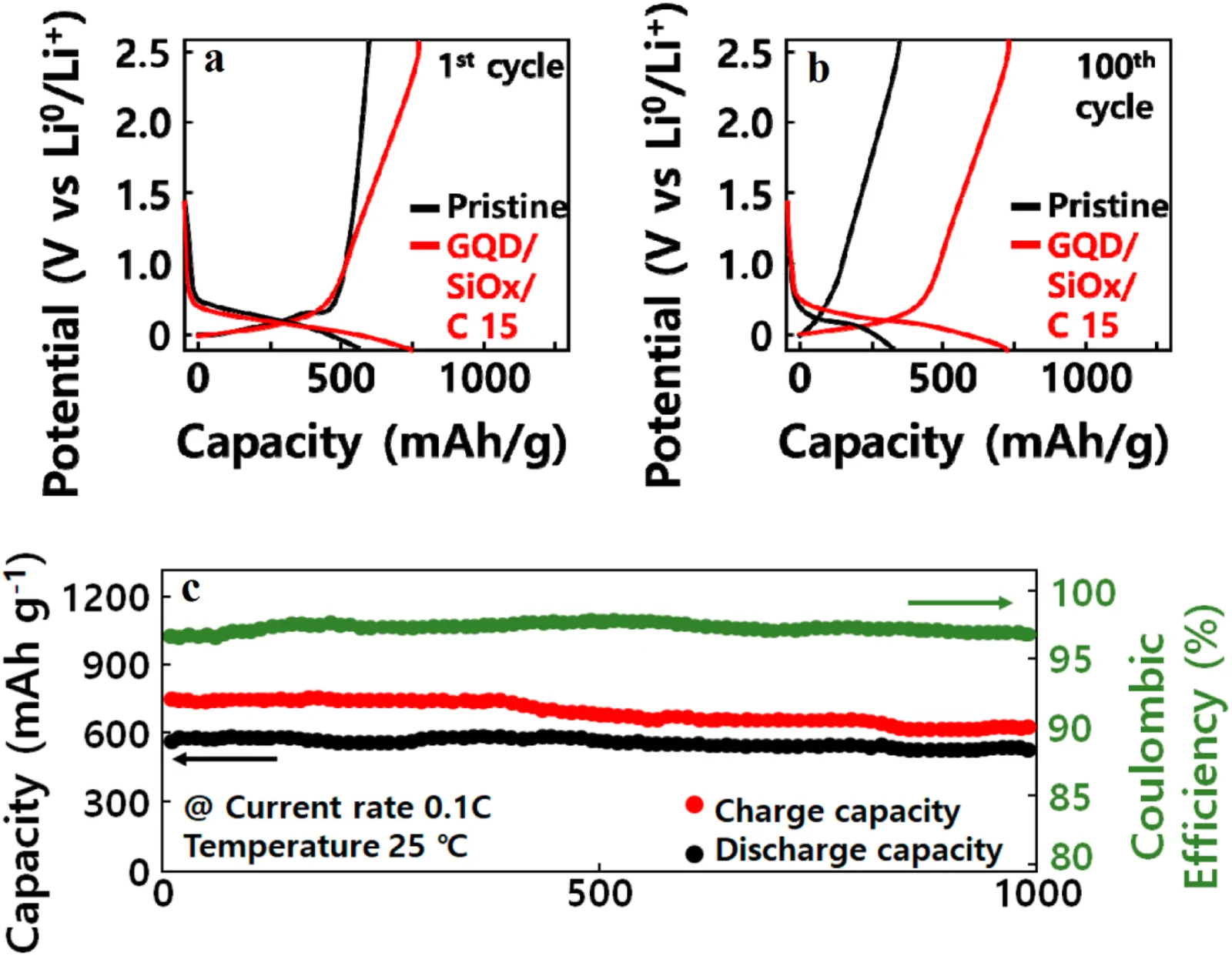
Electrochemical performance and composition-dependent behaviour of GQD/SiO_*x*_/C composite anodes. (a) Coulombic efficiency evolution of GQD/SiO_*x*_/C composites with different SiO_*x*_ contents. (b) Cycling performance of the corresponding composite anodes. (c) Initial charge–discharge profiles of GQD/SiO_*x*_/C composites with different SiO_*x*_ contents. Reproduced from ref. 81 under the Creative Commons Attribution (CC BY) License. © 2024 MDPI.

Fig. 8 summarizes the composition-dependent electrochemical behaviour of GQD/SiO_*x*_/C composite anodes and highlights the importance of balancing lithium-storage capability with structural stability in silicon-derived systems. As shown in Fig. 8(a), the coulombic efficiency evolution demonstrates that introducing an optimized amount of SiO_*x*_ into the GQD/carbon framework improves the reversibility of lithium storage by reducing irreversible reactions during cycling. The enhanced efficiency after the initial cycles suggests that the internal cavity structure generated by GQDs and carbon nanoparticles can accommodate SiO_*x*_-induced volume variation and mitigate continuous electrolyte consumption associated with unstable interfacial reactions. Fig. 8(b) presents the long-term cycling performance of GQD/SiO_*x*_/C composites with different SiO_*x*_ contents, revealing that the capacity improvement depends strongly on composition optimization. Moderate SiO_*x*_ incorporation enhances reversible capacity, whereas excessive SiO_*x*_ loading reduces cycling stability due to insufficient structural buffering space and compromised transport continuity. The initial charge–discharge profiles shown in Fig. 8(c) further confirm the influence of SiO_*x*_ incorporation on lithium-storage behaviour, where increased SiO_*x*_ content provides additional storage sites but requires effective conductive and mechanical support from the surrounding GQD/carbon network. Collectively, these results demonstrate that GQDs contribute not only by improving electronic conductivity but also by constructing an adaptive carbon framework capable of accommodating volume variation, maintaining reaction accessibility, and preserving electrode integrity during prolonged operation.

Quantitative comparison across representative studies further illustrates how the functional role of GQDs depends on the host anode chemistry. In the all-carbon GQD/CNT architecture, reversible capacities of 700 mAh g^−1^ after 100 cycles at 100 mA g^−1^ and 483 mAh g^−1^ after 350 cycles at 1000 mA g^−1^ indicate that combining GQD-derived lithium-interaction sites with a continuous conductive scaffold can support both sustained storage and high-current operation.^74^ In Li_4_Ti_5_O_12_-based systems, the benefit is expressed more clearly through transport and rate metrics: an N-functionalized GQD interfacial layer increased the Li-ion diffusion coefficient by approximately 19% and enabled 161 mAh g^−1^ at 50C for more than 500 cycles,^76^ whereas an N,S-co-doped GQD/LTO framework delivered 254.2 mAh g^−1^ at 0.1C and 126.5 mAh g^−1^ at 10C while retaining at least 96.9% capacity after 2000 cycles at 2C.^77^ For conversion-type CuO, GQD coating increased the capacity after 200 cycles from 61 to 609 mAh g^−1^ and enabled 79.4% retention after 1000 cycles at 2 A g^−1^.^78^ Silicon-based PF-GQD@SiNP electrodes reached 1820 mAh g^−1^ even at 1000 mA g^−1^ and retained 3068 mAh g^−1^ after 100 cycles at 100 mA g^−1^.^80^ These quantitative differences should not be interpreted as a direct ranking because current density, loading, host chemistry, and electrode architecture differ substantially among studies. Rather, they show that the experimentally measurable benefit of GQDs shifts from storage-site utilization in carbon frameworks to transport enhancement, interfacial stabilization, and mechanical-contact preservation in host-dependent systems.

The compiled evidence indicates that the role of GQDs varies substantially with anode chemistry and electrode architecture (Table 3). In all-carbon systems, GQDs can provide additional lithium-interaction sites, whereas Li_4_Ti_5_O_12_-based anodes primarily benefit from enhanced charge transfer, interphase regulation, and high-rate capability. In conversion-type metal oxides, GQD coatings can improve electrical contact and mitigate agglomeration or dissolution, while silicon-based systems depend more strongly on surface functionality, interfacial shielding, and accommodation of volume changes. These differences show that GQD-related performance gains arise from coupled effects rather than from a universal mechanism. For clarity, the unresolved issues in Table 3 are classified into three categories: attribution ambiguity (AA), where the specific GQD contribution cannot be separated from conductive, morphological, or architectural effects; insufficient interfacial/structural characterization (IC), where GQD loading, bonding configuration, interface chemistry, or SEI evolution is inadequately resolved; and practical/long-term validation gaps (PV), including limited cycling, idealized conditions, or insufficient durability assessment. Some systems fall into multiple categories, reflecting the interdependence of mechanistic attribution, structural characterization, and practical stability.

**Table 3:** Comparative battery performance, GQD configurations, functional roles, and mechanistic interpretation of representative GQD-integrated anode systems for lithium-ion batteries

Anode platform/GQD configuration	Battery type	Dominant role of GQDs	Key electrochemical performance	Structure–property–performance interpretation	Main limitation/unresolved issue	Ref.
Freestanding porous CNT sponge coated with functionalized GQDs through electrostatic interaction and π–π stacking	Li-ion battery (LIB)	Li-storage sites; conductive coupling; suppression of GQD aggregation	700 mAh g^−1^ after 100 cycles at 100 mA g^−1^; 483 mAh g^−1^ after 350 cycles at 1000 mA g^−1^	Functionalized GQDs provide Li-storage sites, particularly through oxygen-containing groups, while the 3D CNT framework maintains conductivity and limits GQD agglomeration	AA: contributions from intrinsic GQD lithium storage, the CNT conductive network, interfacial effects, and the freestanding architecture are not quantitatively separated	74
Theoretical GQD models (C_24_H_12_, C_54_H_18_, and C_96_H_24_) under different charge states	LIB-relevant theoretical anode model	Li/Li^+^ adsorption and packing	Li/Li^+^ preferentially adsorb at C_6_ rings near GQD edges; C_24_H_12_ and C_54_H_18_ accommodate up to 4 and 5 Li^+^ ions, corresponding to 0.055 and 0.025 ions per Å^2^	Adsorption depends on GQD size and charge state; increasing Li^+^ loading weakens adsorption because of ion–ion repulsion	PV: the idealized model excludes realistic defects, functional groups, electrolyte interactions, SEI formation, and experimental electrode conditions	75
N-functionalized GQD interfacial layer on Li_4_Ti_5_O_12_	Li-ion battery (LIB)	Charge-transfer mediation; surface protection; SEI regulation; suppression of gassing	∼19% increase in Li-ion diffusion coefficient; 161 mAh g^−1^ at 50C; performance maintained for >500 cycles	The N-functionalized GQD layer acts as a stable charge-transporting and protective interfacial layer while preserving the LTO host as the principal storage phase	AA/IC: the relative contributions of N configuration, GQD loading, conductivity enhancement, and interphase stabilization remain insufficiently separated and structurally resolved	76
Porous Li_4_Ti_5_O_12_ framework hybridized with N,S-co-doped GQDs	Li-ion battery (LIB)	Electronic transport enhancement; additional Li-storage contribution; preservation of electrolyte transport	254.2 mAh g^−1^ at 0.1C; 126.5 mAh g^−1^ at 10C; ≥96.9% capacity retention after 2000 cycles at 2C	N,S-GQDs improve electron transfer and lithium-storage capability, while the interconnected porous LTO framework maintains ionic accessibility and long-term stability	AA/IC: the effects of specific N/S bonding configurations cannot be isolated from those of porosity, morphology, and composite processing	77
Carboxyl-functionalized GQD coating on hierarchical CuO microspheres grown on Cu foam	Li-ion battery (LIB)	Conductivity enhancement; suppression of dissolution/agglomeration; interfacial stabilization	609 mAh g^−1^ after 200 cycles at 0.2 A g^−1^*vs.* 61 mAh g^−1^ for pristine CuO; 79.4% retention after 1000 cycles at 2 A g^−1^; initial coulombic efficiency 88.2%	GQD coating lowers charge-transfer resistance and helps preserve the hierarchical CuO structure while limiting formation of an unstable interfacial layer	AA: contributions from the binder-free architecture, hierarchical morphology, GQD fraction, and coating chemistry are not independently quantified	78
Bottom-up manganese vanadium oxide/GQD nanocomposite	Li-ion battery (LIB)	Nanoscale coupling of redox-active storage and conductive charge-transport domains	∼970 mAh g^−1^ over 100 cycles; coulombic efficiency near 100%	Nanoscale contact between manganese vanadium oxide clusters and GQDs shortens the separation between charge-storage and charge-transport components and improves electrode utilization	IC/PV: GQD mass contribution and interfacial evolution remain insufficiently resolved, while durability beyond 100 cycles has not been established	79
Phenylalanine-functionalized GQD coating on Si nanoparticles (PF-GQD@SiNP)	Li-ion battery (LIB)	Conductivity enhancement; electrolyte shielding; edge-chemistry regulation	4066, 3796, and 1820 mAh g^−1^ at 50, 100, and 1000 mA g^−1^, respectively; 3068 mAh g^−1^ after 100 cycles at 100 mA g^−1^	PF-GQD coating improves electrical conductivity and limits direct Si–electrolyte contact; the phenyl-containing edge structure is associated with improved electron and electrolyte transport relative to AF-GQDs	IC/PV: GQD loading is insufficiently defined, and the limited cycling duration restricts assessment of long-term interfacial and mechanical stability	80
GQD/SiO_*x*_/carbon-nanoparticle composite with composition-dependent internal cavities	Li-ion/lithium secondary battery	Conductive integration; capacity enhancement through SiO_*x*_ incorporation; mechanical buffering by carbon nanoparticles	At 15 wt% SiO_*x*_: initial discharge capacity 595 mAh g^−1^; 92% capacity retention; rate characteristic 81% at 2C relative to 0.1C	SiO_*x*_ increases capacity, while carbon nanoparticles generate internal void space that accommodates volume expansion; the GQD-containing carbon network contributes to electrical connectivity	AA/PV: the specific contribution of GQDs is not isolated from that of carbon nanoparticles, while low initial efficiency and composition-dependent cycling stability remain unresolved	81

### 4.5. Cross-system convergence, evidence gaps, and translational boundaries

When the selected evidence is considered collectively, GQDs emerge less as a single-purpose anode material than as a structurally adaptable nanoscale phase whose dominant function depends on the host environment. The all-carbon studies assign GQDs a partially intrinsic storage role through lithium adsorption, packing, and surface-functionalized binding sites.^74^ By contrast, the lithium titanate systems position them primarily as charge-transporting and interphase-regulating layers,^77^ while the metal-oxide and silicon-derived anodes emphasize conductive coupling, morphological preservation, surface isolation, and mechanical accommodation.^79^ This functional shift across material classes indicates that “GQD enhancement” is not a uniform mechanism. It is an umbrella description for several coupled effects whose relative importance changes with the electronic conductivity, structural stability, surface reactivity, and dimensional response of the host.

A consistent trend nevertheless appears across the evidence: GQDs are most effective when they are placed at an existing performance bottleneck. In poorly conducting but structurally stable hosts, their contribution is associated mainly with charge transfer and interfacial regulation.^76^

Experimentally identifying the dominant performance bottleneck requires combining electrochemical diagnostics with structural and morphological analysis rather than relying on capacity trends alone. Electrochemical impedance spectroscopy can distinguish whether performance is primarily limited by ohmic resistance, interfacial charge transfer, or diffusion-related processes through the evolution of electrolyte resistance, charge-transfer resistance, and low-frequency transport response. Distribution-of-relaxation-time analysis can further separate overlapping kinetic contributions when conventional equivalent-circuit fitting is ambiguous. Cyclic voltammetry at different scan rates can indicate whether charge storage is predominantly diffusion-controlled or surface-dominated, while peak separation and polarization provide additional information on reaction kinetics. GITT or PITT measurements can be used when sluggish Li-ion transport is suspected. Morphological and post-cycling analyses by electron microscopy can identify aggregation, cracking, loss of electrical contact, pore blockage, or unstable surface reconstruction. When combined with XPS, cryo-TEM, or other interphase-sensitive techniques, these measurements can also determine whether SEI growth rather than bulk transport is the dominant limitation. Accordingly, GQD incorporation should be targeted only after the principal kinetic, interfacial, transport, or mechanical bottleneck has been experimentally identified.

In conversion-type oxides, where electrical disconnection, agglomeration, and unstable surface evolution are more severe, GQDs additionally operate as conformal stabilizing phases. In silicon-based systems, the dominant value shifts toward maintaining contact and limiting direct electrolyte exposure during structural variation.^80^ The carbon-nanotube hybrid further shows that intrinsic GQD activity becomes practically accessible only when nanoscale storage sites are integrated into a continuous three-dimensional conductive framework. Theoretical adsorption evidence supports this interpretation but does not independently establish how much reversible capacity survives under realistic surface and electrolyte conditions.^75^

The principal scientific ambiguity is therefore one of attribution. Improvements assigned to GQDs are inseparable from changes in porosity, binder-free construction, host morphology, conductive-scaffold formation, surface functionalization, and composite processing. Even the comparison between phenylalanine- and alanine-functionalized GQDs, which provides relatively direct evidence for the importance of edge chemistry, simultaneously changes steric organization and energy-level distribution. Likewise, the effects of nitrogen functionalization and N,S co-doping cannot be ranked independently because their host architectures and incorporation modes differ.^67,76^ The literature consequently establishes correlation more convincingly than isolated causation.

Translation beyond laboratory-scale electrodes is further constrained by the diversity of fabrication routes. *In situ* deposition, microwave-assisted hybridization, hydrothermal coating, bottom-up cluster conversion, molecular functionalization, and multicomponent mixing impose different requirements for temperature, solvent use, compositional control, and coating uniformity.^57,79^ These differences complicate reproducibility and make performance comparisons sensitive to processing history. Moreover, the supplied studies predominantly emphasize gravimetric capacity and cycling behaviour, whereas GQD loading, electrode density, areal performance, interfacial mass, and the fraction of inactive components are not consistently resolved.

The convergent design implication is that GQDs should be assigned a clearly defined role before composite optimization: direct storage site, electronic bridge, interphase regulator, protective coating, or mechanical-contact stabilizer. Future architectures derived from this evidence should minimize unnecessary GQD content, preserve ion-accessible pathways, and employ control electrodes capable of separating chemical, architectural, and conductive contributions. Overall, the selected studies confirm the versatility of GQDs but also reveal that mechanistic attribution, scalable processing, and electrode-level comparability remain insufficiently standardized.

The structural-reporting limitations of the representative studies considered in this review also constrain mechanistic interpretation. In the N-functionalized and N,S-co-doped Li_4_Ti_5_O_12_ systems, the electrochemical consequences of heteroatom incorporation are evident, but the relative abundance and spatial distribution of specific nitrogen- and sulfur-bonding configurations are not sufficiently resolved to identify the dominant atomic motifs. In the manganese vanadate/GQD composite, GQD mass fraction, interfacial evolution, and the structural state of the GQD component during cycling remain insufficiently quantified.^79^ For the functionalized GQD/Si system, the unspecified GQD loading limits assessment of whether the observed behaviour originates from edge chemistry, coverage, or changes in conductive connectivity.^80^ In the GQD/SiO_*x*_/carbon-nanoparticle composite, the specific structural and electrochemical contribution of the GQD phase is not isolated from that of the accompanying carbon nanoparticles.^81^ More generally, several representative studies provide average particle dimensions or elemental composition without simultaneously resolving thickness distribution, sp^2^-domain coherence, edge-to-basal-plane structure, dopant bonding configuration, aggregation state, or post-cycling structural evolution. These omissions do not invalidate the reported electrochemical improvements, but they reduce confidence in assigning those improvements to a specific GQD structure or mechanism.

## 5. Critical challenges, mechanistic uncertainties, and future design roadmap

### 5.1. Resolving dynamic storage mechanisms and interphase-coupled reactivity

The principal mechanistic uncertainty in GQD-integrated anodes is not whether GQDs alter electrochemical behaviour, but which atomic and interfacial processes dominate under operating conditions. Lithium may interact with basal-plane defects, edge states, heteroatomic sites, oxygen-containing groups, confined pores, or electronically polarized interfaces. These contributions can coexist and evolve during cycling, making capacity attribution from *ex situ* structural characterization inherently ambiguous. In particular, adsorption at surface sites, intercalation-like accommodation, pseudocapacitive storage, electrolyte-derived reactions, and reversible interphase participation may generate overlapping electrochemical signatures. Treating excess capacity as direct evidence of additional GQD storage sites therefore risks conflating distinct mechanisms.

A more reliable framework requires time-resolved correlation between structural state, local bonding, charge distribution, and electrochemical response. *Operando* Raman spectroscopy can track disorder and conjugation changes, while synchrotron-based absorption and photoelectron methods can resolve changes in dopant coordination and oxidation state. Solid-state nuclear magnetic resonance, isotope-labelled electrolytes, differential electrochemical mass spectrometry, and electrochemical quartz-crystal measurements can help distinguish lithium accommodation from electrolyte decomposition and interphase growth.^74,81^ These techniques should be integrated with kinetic analysis rather than applied as isolated characterization tools. Rate-dependent cyclic voltammetry, intermittent titration methods, impedance evolution, and distribution-of-relaxation-time analysis can separate transport limitations from changes in the number or accessibility of reactive sites.

A concrete experimental strategy for resolving capacity attribution is to construct matched GQD libraries in which only one structural variable is systematically changed while the remaining descriptors are held as constant as experimentally possible. For example, GQDs with comparable lateral size, thickness, oxygen content, loading, and electrode porosity could be prepared with different edge structures to evaluate the contribution of edge-derived lithium storage. Likewise, nitrogen-doped GQDs with similar total N content but different relative fractions of pyridinic, pyrrolic, and graphitic N would allow configuration-dependent lithium interaction and charge-transfer effects to be compared without conflating them with overall dopant concentration. A parallel series with controlled oxygen-functional-group densities could isolate the contribution of oxygenated sites. These matched samples should be tested using identical electrode formulations and electrochemical protocols and compared through differential capacity analysis, scan-rate-dependent cyclic voltammetry, GITT/PITT, impedance spectroscopy, and *operando* or post-cycling spectroscopic characterization. Isotope-labelled electrolytes or solid-state NMR could further determine whether additional capacity originates from reversible lithium accommodation or electrolyte-derived interphase products. Such one-variable-at-a-time comparative designs would provide a more defensible route for assigning capacity to specific GQD configurations rather than to correlated changes in morphology, conductivity, or interphase chemistry.

Theoretical modelling must also move beyond idealized, isolated GQD structures. Mechanistic predictions should incorporate realistic edge disorder, mixed functional groups, solvent coordination, surface charge, interfacial polarization, and evolving interphase fragments. Constant-potential calculations and reactive molecular simulations are particularly relevant because the electronic state of the electrode changes continuously during operation.

The central objective is to establish configuration-specific mechanisms rather than assign universal behaviour to all GQDs.

A more decisive experimental strategy should combine matched electrode controls with time-resolved structural and electrochemical measurements. At minimum, a GQD-containing electrode should be compared with the host-only electrode, an electrode containing an equivalent mass fraction of non-quantized conductive carbon, and, where relevant, GQDs with systematically varied surface chemistry but matched particle loading and electrode porosity. Such controls can help separate capacity gains caused by additional conductive pathways from those associated with GQD-specific storage or interfacial chemistry. Isotopic tracing provides a complementary route: isotope-labelled electrolyte components or lithium sources, coupled with solid-state NMR or mass-spectrometric analysis, can help distinguish lithium accommodated within carbonaceous domains from species incorporated into electrolyte-derived interphase products. *Operando* Raman, X-ray absorption, or photoelectron measurements can then track changes in graphitic order, bonding configuration, oxidation state, and interphase chemistry during lithiation and delithiation. Electrochemical analyses should be applied in parallel. GITT or PITT can quantify changes in apparent Li-ion transport, EIS and distribution-of-relaxation-time analysis can separate evolving charge-transfer and interphase resistances, and scan-rate-dependent cyclic voltammetry can estimate the relative contributions of diffusion-controlled and surface-dominated charge storage. EQCM or differential electrochemical mass spectrometry can further identify mass changes or gaseous products associated with electrolyte decomposition. No single technique can uniquely assign excess capacity to GQDs; rather, attribution requires convergence among matched controls, isotopic or chemical tracing, *operando* characterization, and kinetic analysis. Such a multimodal protocol would provide a substantially stronger basis for distinguishing intrinsic GQD lithium storage from conductivity enhancement and interphase-mediated contributions.

### 5.2. Minimum characterization requirements, descriptor standardization, and reproducible electrochemical benchmarking

Progress in GQD-based anodes is constrained by inconsistent material definitions and incomplete reporting. Lateral size alone cannot adequately represent a GQD population because electronic and interfacial behaviour also depends on thickness, size distribution, edge topology, crystallinity, sp^2^-domain continuity, defect density, surface composition, dopant configuration, aggregation state, and colloidal stability. Consequently, materials described using the same GQD label may possess substantially different active-site distributions, electronic structures, and transport properties. Without a standardized structural identity, reported performance differences cannot be reliably linked to specific design variables.

A practical minimum characterization set should therefore be satisfied before a carbon nanomaterial is classified as a GQD. TEM should be used to establish nanoscale lateral dimensions and morphology, supported by a statistically meaningful particle-size distribution rather than selected representative images. HRTEM should additionally be used, where feasible, to verify locally ordered graphene-like lattice domains and to distinguish structurally coherent nanographene regions from predominantly amorphous carbon. AFM should provide particle-height and thickness distributions and, where possible, information related to layer number. Raman spectroscopy should report the D and G bands, including their positions, widths, and the *I*_D_/*I*_G_*I*_D_/*I*_G_ ratio, but this ratio should not be interpreted independently as a direct measure of either sp^2^ fraction or crystallinity. Raman results should instead be considered together with microscopic or diffraction evidence. XPS should quantify elemental composition and resolve the major carbon bonding environments, including sp^2^/sp^3^-related components and relevant oxygen- or heteroatom-containing configurations. For doped GQDs, total heteroatom content alone is insufficient; graphitic, edge-bound, oxidized, and functional-group-associated states should be differentiated where experimentally resolvable.

Ensemble-level characterization is equally important. Size distribution, thickness distribution, aggregation state, and, where relevant, colloidal stability should be reported statistically because mean values can conceal chemically or structurally distinct particle subpopulations. Batch-to-batch variation should also be evaluated, particularly when synthesis involves multiple purification or functionalization steps.^75,80^ Accordingly, nanoscale size or fluorescence alone should not be regarded as sufficient evidence for GQD identity. A defensible classification should be supported by converging evidence for a confined, structurally coherent sp^2^-rich nanographene domain, finite thickness, and a chemically resolved surface structure.

Electrochemical benchmarking requires similar discipline. Gravimetric capacity should be accompanied by areal capacity, electrode density, active-material loading, porosity, thickness, initial coulombic efficiency, voltage profile, current normalization, and the fractions of binder and conductive additive. Rate capability should be evaluated under controlled loading and electrode geometry, while long-term cycling should report capacity retention, coulombic efficiency, impedance evolution, and clearly defined failure criteria. Half-cell data alone cannot establish practical relevance because excess counter-electrode lithium can mask irreversible losses and unstable interphase chemistry. Where possible, full-cell validation under realistic negative-to-positive capacity balancing and limited electrolyte conditions should therefore complement half-cell measurements.

Control designs are equally important for separating GQD-specific effects from architectural or processing contributions. Comparisons should isolate the influence of GQD chemistry, GQD loading, host morphology, conductive scaffolds, and processing conditions. Appropriate controls include undoped and differently functionalized GQDs, equivalent non-quantized carbon additives, host-only electrodes, and composites with matched carbon content and porosity. Standardized structural descriptors, minimum characterization criteria, electrochemical reporting protocols, and matched controls would convert isolated performance reports into more testable structure–mechanism relationships. Once comparability is established, the next challenge is preserving these relationships during scalable production and electrode fabrication.

Table 4 converts the multidimensional definition of GQDs into a practical minimum reporting framework. Not every technique is required in every study, but classification as a GQD and subsequent mechanistic claims should be supported by converging evidence for lateral confinement, finite thickness, coherent sp^2^-rich nanographene structure, chemically resolved surface composition, and statistically characterized heterogeneity. Additional descriptors should be reported whenever performance is attributed specifically to dopant configuration, edge states, defects, or interphase regulation. This checklist is intended to improve cross-study comparability without imposing a single synthesis- or application-specific definition of GQDs.

**Table 4:** Minimum reporting checklist for structural identification and electrochemical evaluation of GQDs used in lithium-ion battery anodes

Reporting category	Minimum information to report	Preferred characterization	Why it is required
Lateral dimensions	Mean size, distribution, number of particles analysed	TEM + statistical size analysis	Avoids classification based on selected images
Thickness/layer structure	Height distribution and approximate layer number	AFM; complementary HRTEM where appropriate	Distinguishes few-layer GQDs from thicker graphitic nanoparticles
Nanographene order	Presence and continuity of graphene-like domains	HRTEM; Raman; diffraction where applicable	Supports assignment of a coherent sp^2^ nanographene structure
Raman structure	D/G positions, widths and *I*_D_/*I*_G_*I*_D_/*I*_G_	Raman spectroscopy	Provides comparative information on disorder and finite-domain structure; *I*_D_/*I*_G_*I*_D_/*I*_G_ should not be used alone as a crystallinity metric
Carbon bonding	sp^2^/sp^3^-related bonding environments	High-resolution XPS C 1s	Establishes the degree of preserved π-conjugated carbon
Surface chemistry	Major oxygen-containing and other functional groups	XPS; FTIR where useful	Enables correlation of surface chemistry with Li interaction and SEI formation
Dopant configuration	Total content plus pyridinic, pyrrolic, graphitic, oxidized or other resolvable states	High-resolution XPS; complementary spectroscopy where available	Total dopant concentration cannot identify the active atomic configuration
Edge/defect information	Edge-rich character, vacancy/defect indicators, where experimentally accessible	Raman, HRTEM and complementary spectroscopy	Required before assigning performance to edge or defect sites
Ensemble heterogeneity	Size/thickness distribution, aggregation and colloidal stability	TEM/AFM statistics, DLS where appropriate	Mean values can conceal structurally distinct populations
GQD loading	GQD mass fraction or surface coverage in the composite	Gravimetric/compositional analysis	Necessary to compare performance and separate GQD from host contributions
Electrode parameters	Loading, thickness, density, porosity, binder and conductive-additive fractions	Standard electrode reporting	Required for meaningful cross-study electrochemical comparison
Interphase evolution	SEI composition/morphology before and after cycling where mechanistic claims are made	XPS, cryo-TEM, TOF-SIMS, *operando*/*in situ* methods	Tests claims of GQD-mediated interphase stabilization
Reproducibility	Batch-to-batch structural descriptors and electrochemical variation	Replicate synthesis and testing	Establishes whether the reported GQD identity is reproducible

### 5.3. Scalable GQD production and manufacturing-compatible electrode integration

Laboratory synthesis often prioritizes tunability, whereas practical manufacturing requires consistency, yield, cost control, environmental compatibility, and compatibility with existing electrode-processing infrastructure. GQD production commonly involves oxidative cutting, hydrothermal conversion, microwave treatment, electrochemical fragmentation, or bottom-up carbonization. Each route generates a distinct distribution of sizes, functional groups, residual precursors, inorganic contaminants, and structural disorder. Scaling these routes is therefore not a simple increase in batch volume. Heat and mass transfer, reaction gradients, purification efficiency, and precursor variability can alter the atomic configurations responsible for the intended function.

Manufacturing-oriented synthesis should be designed around measurable critical quality attributes. These include yield, particle-size distribution, and colloidal stability, dopant bonding configuration, surface-group density, residual ion content, and electronic conductivity after drying. Continuous-flow and modular processing may provide tighter residence-time and temperature control than batch synthesis, but only when coupled with inline monitoring. Optical spectroscopy, conductivity measurements, particle-size analysis, and automated chemical assays could be incorporated as process-control tools, provided that they are calibrated against configuration-sensitive structural measurements.^76,79^

Purification presents a separate constraint. Dialysis and repeated centrifugation can produce clean laboratory samples but may be difficult to translate economically. Residual acids, salts, molecular fluorophores, and unreacted precursors can alter interphase chemistry and create misleading performance gains or losses. Solvent recovery, water consumption, precursor toxicity, and waste generation should therefore be considered alongside electrochemical performance. Sustainable precursor selection is valuable only when the complete synthesis and purification burden is evaluated.

Electrode integration also requires attention to slurry rheology, drying behaviour, binder compatibility, agglomeration, and adhesion. Highly functionalized GQDs may disperse well in polar media but interact unfavourably with binders or absorb excessive electrolyte. Conversely, reduced GQDs may provide better conductivity while forming aggregates during mixing and drying. The optimum formulation must preserve nanoscale distribution after calendaring and under practical electrode thickness. Scalable synthesis and processing must therefore be co-developed rather than optimized independently. This manufacturing perspective leads directly to the need for a coordinated roadmap linking material discovery with cell-level validation.

### 5.4. Closed-loop design, near-term application scenarios, and translation to practical cells

The next generation of GQD-enabled anodes should be developed through closed-loop design rather than sequential trial-and-error modification. Such a framework begins with a predefined functional objective, such as increasing electronically accessible reaction zones, regulating interphase chemistry, maintaining contact during dimensional change, or reducing transport heterogeneity. GQD size, edge composition, dopant configuration, loading, and spatial placement should then be selected according to that objective. This role-based strategy is more transferable than maximizing a single property such as surface area, defect density, or heteroatom content.

Multiscale modelling can support this transition by connecting atomistic descriptors to electrode-level behaviour. Electronic-structure calculations can identify configuration-dependent binding and charge-transfer tendencies; molecular simulations can examine solvent organization and early interphase reactions; mesoscale models can describe aggregation, pore evolution, and conductive-network formation; and continuum models can predict concentration gradients, local current density, and stress in thick electrodes. The principal challenge is passing physically meaningful parameters between these scales rather than treating each model independently.^77,81^

Data-assisted methods can accelerate this process when trained on standardized, chemically resolved datasets. Useful inputs should include bonding configurations, functional-group distributions, synthesis conditions, electrode composition, loading, density, and testing protocols rather than broad labels such as “N-doped GQD.” Model outputs should target mechanistically meaningful responses, including irreversible lithium loss, interphase resistance growth, transport heterogeneity, and retention of conductive connectivity. Interpretability and uncertainty estimation are necessary because correlations derived from small, heterogeneous datasets can otherwise be mistaken for design laws.

Translation must culminate in practical validation. Promising materials should progress from controlled half-cells to full cells with realistic capacity balancing, limited electrolyte, industrially relevant loading, appropriate formation protocols, and evaluation across temperature and fast-charging conditions. Safety, self-discharge, gas generation, storage stability, and post-cycling failure analysis should be considered alongside capacity retention. The roadmap is therefore not a search for a universally optimal GQD, but a disciplined process for matching a reproducible GQD configuration to a defined electrode limitation and verifying that benefit under practical constraints. These requirements frame the broader conclusions of this review.

From a near-term application perspective, the most credible opportunities for GQD integration are likely to arise where the GQDs address a clearly defined limitation of an already established host anode rather than functioning as an independent high-capacity storage phase. Li_4_Ti_5_O_12_-based anodes represent one such scenario because GQD interfacial layers have already demonstrated improvements in high-rate charge transfer, Li-ion transport, surface protection, and prolonged cycling without requiring replacement of the structurally stable host. Such architectures may therefore be particularly relevant to battery systems prioritizing power capability, rapid charging, long cycle life, and interfacial stability. Silicon and SiO_*x*_-based composites constitute a second promising direction, where the principal near-term value of GQDs is likely to lie in maintaining electronic contact, regulating electrolyte exposure, and stabilizing interfaces during repeated dimensional changes rather than directly maximizing lithium-storage capacity. GQD-modified conversion-type metal oxides may provide a further opportunity through conformal conductive coupling and suppression of agglomeration or surface degradation, although their more extensive structural reconstruction introduces additional uncertainty.

Commercial implementation nevertheless requires several technical barriers to be resolved across these scenarios. GQD synthesis must deliver reproducible particle size, surface chemistry, dopant configuration, purity, and yield at manufacturing scale, while purification and functionalization should not impose excessive solvent, water, energy, or processing burdens. Electrode fabrication must preserve nanoscale GQD distribution during slurry mixing, drying, and calendaring without compromising binder compatibility, packing density, electrolyte accessibility, or electrode thickness. The optimum GQD loading must also be minimized and quantitatively justified because excessive incorporation can increase surface reactivity, electrolyte consumption, inactive mass, or aggregation. At the cell level, performance advantages should persist under practical areal loading, limited electrolyte, realistic negative-to-positive capacity balancing, and full-cell operation rather than only in lithium-metal half-cells. Initial coulombic efficiency, interphase resistance growth, gas generation, safety, storage stability, and long-term mechanical integrity must be evaluated alongside gravimetric capacity. Accordingly, the most realistic commercialization pathway is not the universal replacement of conventional anodes by GQDs, but the targeted use of reproducible, low-loading GQD interfacial or conductive modifiers where their performance benefit can be demonstrated under manufacturing-relevant conditions.

### 5.5. Physical, experimental, and economic constraints toward practical GQD anodes

Beyond mechanistic uncertainty and synthesis reproducibility, the practical use of GQDs in lithium-ion battery anodes is constrained by a combination of physical, experimental, and economic factors. At the physical level, the nanoscale dimensions and high surface-to-volume ratio of GQDs are advantageous for interfacial contact but can also increase their tendency to aggregate, particularly during drying, slurry mixing, and electrode calendaring. Excessive GQD loading may reduce pore accessibility, increase tortuosity, or lower electrode packing density, thereby offsetting gains in electronic connectivity. Highly functionalized GQDs may additionally increase electrolyte uptake and interfacial reactivity, potentially promoting irreversible lithium consumption and nonuniform interphase formation. These effects become increasingly important in thick electrodes, where local GQD distribution, conductive percolation, mechanical adhesion, and preservation of ion-accessible pathways must be maintained simultaneously. Thus, the physical benefit of GQDs depends not only on their intrinsic properties but also on whether their nanoscale function survives electrode-scale processing and cycling.

Experimental constraints further complicate performance assessment. Many reported GQD-integrated anodes are evaluated under conditions that are difficult to compare directly because active-material loading, electrode thickness, current normalization, electrolyte amount, porosity, and conductive-additive content are not consistently reported. Half-cell configurations also provide limited evidence of practical performance because excess lithium from the counter electrode can mask irreversible lithium loss and interphase instability.^76,79^ Reliable evaluation therefore requires matched control electrodes, standardized mass loading, areal-capacity reporting, impedance evolution, and full-cell testing under realistic negative-to-positive capacity ratios. *Operando* or time-resolved characterization is also needed to determine whether improvements arise from direct lithium storage, interfacial stabilization, conductive-network formation, or morphology preservation rather than from changes in electrode architecture alone.

Economic constraints represent a separate translational barrier. Laboratory-scale GQD synthesis frequently relies on hydrothermal treatment, microwave processing, oxidative cutting, functionalization, dialysis, centrifugation, and repeated washing. Although these approaches can provide well-controlled laboratory materials, their cost can increase substantially when solvent consumption, water use, purification time, precursor utilization, waste treatment, and quality-control requirements are considered. Purification is particularly important because residual acids, salts, molecular by-products, or unreacted precursors may alter electrochemical behaviour, yet removing these impurities at large scale can be resource-intensive. In addition, the economic value of adding GQDs must be assessed against simpler conductive carbons or surface-coating strategies that may provide comparable gains at lower processing complexity. Practical deployment therefore requires not only high electrochemical performance but also high-yield synthesis, reproducible quality attributes, low purification burden, compatibility with established electrode manufacturing, and a demonstrable performance benefit per unit processing cost.

## 6. Conclusion

GQDs introduce a distinctive design space for lithium-ion battery anodes because their electronic structure, edge chemistry, surface states, and nanoscale dimensions can be coupled directly to host materials and electrode architectures. Their value, however, cannot be reduced to a universal enhancement mechanism. Depending on their configuration and placement, GQDs may provide lithium-interaction sites, redistribute interfacial charge, regulate interphase development, bridge conductive discontinuities, or preserve contact within structurally evolving electrodes. The central contribution of this review is therefore a multiscale framework that connects structural identity to functional descriptors, dynamic interfaces, coupled transport, and electrode-level translation. The major limitation of the field is insufficient mechanistic attribution. Performance improvements are frequently inseparable from changes in porosity, morphology, carbon content, processing, and electrode architecture.

Progress requires configuration-resolved characterization, realistic *operando* analysis, matched control electrodes, and standardized reporting that extends beyond gravimetric capacity. At the same time, synthesis and purification must be redesigned around reproducibility, manufacturing compatibility, and measurable quality attributes. The most credible route forward is role-based rather than additive-based design. GQDs should be engineered for a predefined bottleneck, incorporated at the location where that function is required, and validated under practical full-cell conditions. This transition from empirical incorporation to descriptor-guided, failure-aware integration will determine whether GQDs develop from versatile laboratory modifiers into reproducible components of advanced anode systems.

## Conflicts of interest

There are no conflicts to declare.

## Data Availability

No primary research results, software or code have been included and no new data were generated or analysed as part of this review.
